# Some dipole shower studies

**DOI:** 10.1140/epjc/s10052-018-5645-z

**Published:** 2018-03-16

**Authors:** Baptiste Cabouat, Torbjörn Sjöstrand

**Affiliations:** 1 Theoretical Particle Physics, Department of Astronomy and Theoretical Physics, Lund University, 223 62, Lund, Sweden; 2 School of Physics and Astronomy, University of Manchester, Schuster Building, Oxford Road, Manchester, M13 9PL UK

## Abstract

Parton showers have become a standard component in the description of high-energy collisions. Nowadays most final-state ones are of the dipole character, wherein a pair of partons branches into three, with energy and momentum preserved inside this subsystem. For initial-state showers a dipole picture is also possible and commonly used, but the older global-recoil strategy remains a valid alternative, wherein larger groups of partons share the energy–momentum preservation task. In this article we introduce and implement a dipole picture also for initial-state radiation in Pythia, and compare with the existing global-recoil one, and with data. For the case of Deeply Inelastic Scattering we can directly compare with matrix element expressions and show that the dipole picture gives a very good description over the whole phase space, at least for the first branching.

## Introduction

In the current description of high-energy collisions, such as those at the LHC, parton showers play a key role [1, 2]. The natural starting point for a description of the perturbative stage of the collisions is to use matrix-element (ME) calculations, but with increasing parton multiplicity these rapidly become quite time-consuming. A practical limit lies around eight final-state partons for leading-order (LO) calculations and four for next-to-leading-order (NLO) ones. By contrast, a high-$p_{\perp }$ LHC collision could contain a hundred partons above a 1 GeV lower cutoff scale. It is therefore natural to combine the ME calculations for a few energetic and well separated partons with the parton-shower ones, that in an approximate manner can add further soft and collinear emissions.

The concept of parton showers is implicit already in the DGLAP evolution equations [3–5], and over the years many shower algorithms have been written. In its simplest incarnation, a shower implements a set of successive partonic branchings $a \rightarrow b + c$, where the two daughters *b* and *c* can branch further in their turn. Showers may differ in a number of respects, such as how emissions are ordered by an evolution variable, how energy and momentum is shared between the daughters of a branching, and how overall energy and momentum conservation is ensured. It is also necessary to distinguish between initial-state radiation (ISR) and final-state radiation (FSR), where the former involves a succession of spacelike partons stretching from the original incoming protons to the hard interaction, while the latter describes a cascade of timelike partons occurring afterwards. The naive choice of evolution variable, to order possible emissions, is the spacelike or timelike virtuality *Q* of a parton, since by Heisenberg’s uncertainty relation the proper lifetime of it should be of order 1 / *Q* (for $\hbar = 1$), such that lower *Q*’s should correspond to earlier times for ISR and later for FSR.

The virtuality choice does not take into account the possibility of destructive interference in the soft-gluon radiation pattern surrounding a pair of colour-correlated hard partons, however. This can be solved by instead evolving in terms of a gradually decreasing emission angle [6, 7]. With modest updates [8] this algorithm remains the default in the Herwig event generator [9, 10], and has been successful over the years. To note is that the algorithm is not completely Lorentz-frame-independent and that overall energy–momentum conservation is only ensured at the very end by some nontrivial transformations.

**Fig. 1:**
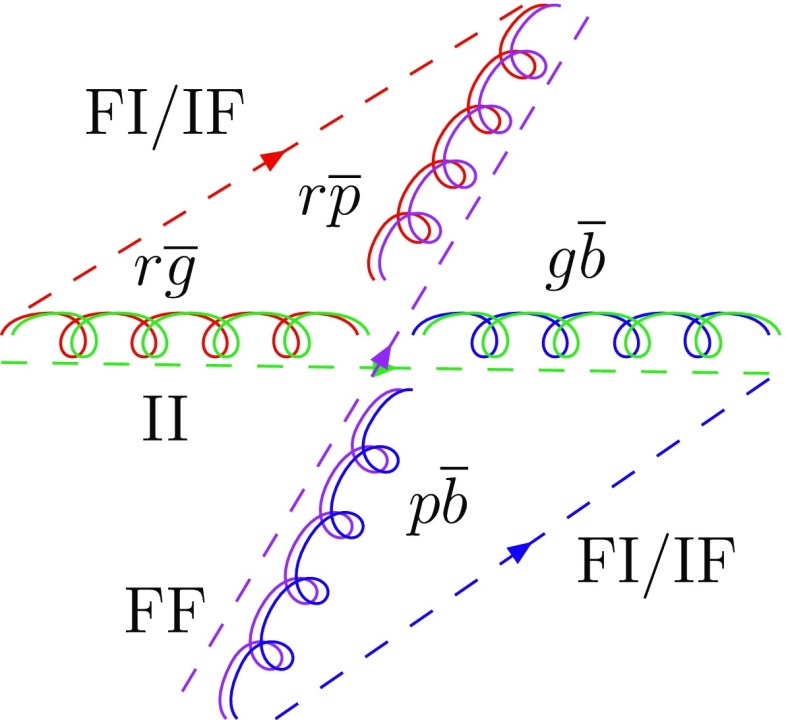
Colour flow for the process $\mathrm {g}(r\overline{g}) + \mathrm {g}(g\overline{b}) \rightarrow \mathrm {g}(r\overline{p}) + \mathrm {g}(p\overline{b})$. Here, the $N_{\mathrm {C}} \rightarrow \infty$ limit is used so that *p* stands for the new colour purple. The dashed lines represent the colour lines stretching between the dipole ends. The type of dipole is indicated

An alternative is the dipole approach [11], first implemented in the Ariadne algorithm [12, 13], which also achieves a correct handling of soft-gluon interference aspects. In it the $1 \rightarrow 2$ branching paradigm is replaced by a $2 \rightarrow 3$ one, where the original dipole is defined by a pair of matching colour–anticolour partons, as defined in the $N_{\mathrm {C}} \rightarrow \infty$ limit [14], where each colour label is unique. Often it is convenient to split the full radiation pattern into two dipole-end contributions, where one of the two partons acts as radiator and the other as recoiler, with four-momentum preserved inside the dipole. The terminology then is to refer to FF, II, FI and IF emissions, depending on whether the radiator and recoiler are in the final (F) or initial (I) state. The FI and IF cases occur when a colour line flows from the initial to the final state. An example of every type of dipoles is given in Fig. 1. The dipole approach is, in many variants, standard in generators such as Sherpa [15–17], Vincia [18, 19] and Dire [20], and is an option in Herwig [21]. For the extensions to ISR, often the Catani–Seymour dipole kinematics is used [22].

**Fig. 2:**
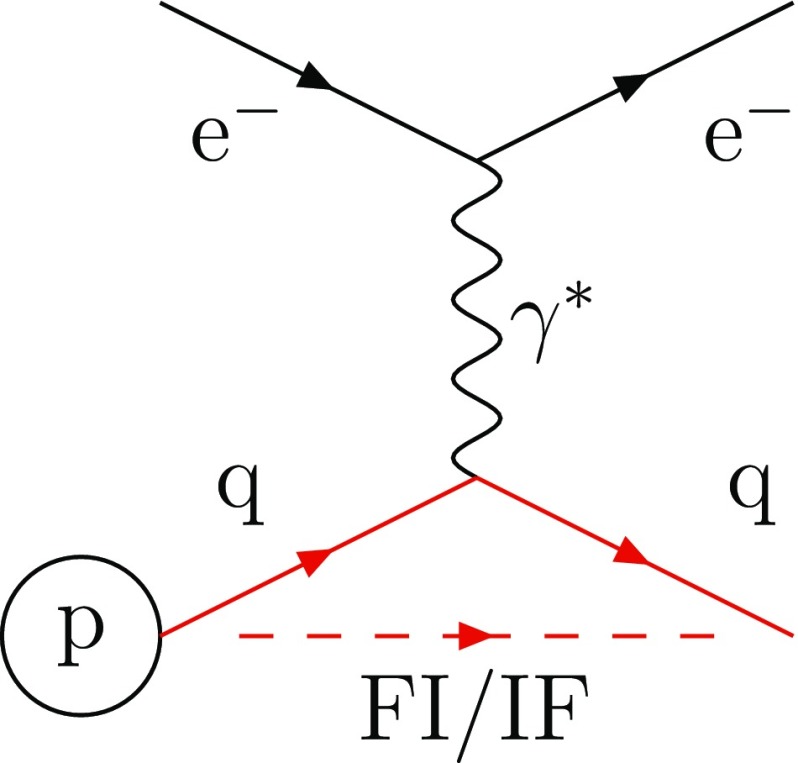
Deeply inelastic scattering: an incoming electron scatters one of the quark within the incoming proton. The dashed line represents the colour line stretching between the two dipole ends

The Pythia generator [23–25], is also dipole-based for FSR, both FF and FI topologies, but ISR is implemented in the so-called global recoil scheme that is implicit in an II dipole setup, wherein all final-state particles share the recoil of an ISR emission. This is a perfectly valid approach for a process like $\gamma ^* / {\mathrm {Z}^0}$ production at hadron colliders, insofar as it attaches well with a ME-based view of the production process. A consistent FI/IF dipole handling is essential for a description of showers in Deeply Inelastic Scattering (DIS), however [20, 26]. For this case, it can be seen in Fig. 2 that a FI/IF dipole naturally stretches between the incoming quark and the final scattered quark. In the current article, therefore, we develop and implement a description of the IF emission topology, and combine it with the FI contribution. As it turns out, it is possible to set up kinematics such that the IF contribution matches the DIS gluon-emission ME, thereby providing an economical description. The new framework also allows a comparison of dipole vs. global recoil e.g. for $\gamma ^* / {\mathrm {Z}^0}$ production at hadron colliders.

Do note that the showers described in this article are formally accurate only to leading log (LL), although many aspects of next-to-leading-log (NLL) physics are implemented, such as the use of $p^2_{\perp }$ as $\alpha _{\mathrm {s}}$ scale. By contrast, while early attempts to develop NLL parton showers petered out (NLLJET [27]), the main thrust of current shower development is in that direction, with first implementations in Vincia [28] and Dire [29, 30]. An important aspect of this game is also to correctly include subleading colour corrections [31].

The plan of the article is as follows. In Sect. 2 we describe the current default framework for ISR and FSR in Pythia, to set the stage. Section 3 introduces the new alternative framework for the $\hbox {FI} + \hbox {IF}$ dipole handling, with special emphasis on the comparison with the DIS MEs. A first comparison with data is presented in Sect. 4, both for DIS and for $\gamma ^* / {\mathrm {Z}^0}$ and jets production at hadron colliders. Finally Sect. 5 provides a summary and outlook.

## The existing framework

Given a hard process as starting point, Pythia will create a parton-level event by interleaving ISR, FSR and MPI (multiparton interaction) activity in a combined downwards evolution in transverse momentum

1 $$\begin{aligned} \displaystyle \frac{\mathrm {d}\mathscr {P}}{\mathrm {d}p_{\perp }}= & {} \displaystyle \left( \frac{\phantom {\left( \right) } \mathrm {d}\mathscr {P}_{\mathrm {MPI}}}{\mathrm {d}p_{\perp }} + \sum \frac{\phantom {\left( \right) } \mathrm {d}\mathscr {P}_{\mathrm {ISR}}}{\mathrm {d}p_{\perp }} + \sum \frac{\phantom {\left( \right) } \mathrm {d}\mathscr {P}_{\mathrm {FSR}}}{\mathrm {d}p_{\perp }} \right) \nonumber \\&\displaystyle \times \exp \left( - \int _{p_{\perp }}^{p_{\perp \mathrm {max}}} \left( \frac{\phantom {\left( \right) } \mathrm {d}\mathscr {P}_{\mathrm {MPI}}}{\mathrm {d}p_{\perp }'} \right. \right. \nonumber \\&\displaystyle \left. \left. + \sum \frac{\phantom {\left( \right) } \mathrm {d}\mathscr {P}_{\mathrm {ISR}}}{\mathrm {d}p_{\perp }'} + \sum \frac{\phantom {\left( \right) } \mathrm {d}\mathscr {P}_{\mathrm {FSR}}}{\mathrm {d}p_{\perp }'} \right) \mathrm {d}p_{\perp }' \right) ~, \end{aligned}$$

that probabilistically determines what the next step will be [32]. Here the ISR sum runs over all incoming partons, two for each already produced MPI, including the hard interaction itself, the FSR sum runs over all outgoing partons, and $p_{\perp \mathrm {max}}$ is the $p_{\perp }$ of the previous step. The Sudakov-style [33] exponential ensures that probabilities are bounded by unity. While FSR is described by evolution from the hard process forwards, ISR is described by evolution from it backwards to the shower initiators [34]. The decreasing $p_{\perp }$ scale therefore is not a simple time variable, but can instead be viewed as an evolution towards increasing resolution power. That is, given that the event has a particular structure when activity above some $p_{\perp }$ scale is resolved, how might that picture change when the resolution cutoff is reduced by some infinitesimal $\mathrm {d}p_{\perp }$?

The ISR and FSR branching probabilities in Eq. (1) are provided by standard DGLAP evolution equations, where the evolution variable is a modified $p_{\perp }$ scale

2 $$\begin{aligned} p^2_{\perp \mathrm {evol}}= & {} z (1 - z) Q^2 ~~ \mathrm {for~FSR} ~, \end{aligned}$$

3 $$\begin{aligned} p^2_{\perp \mathrm {evol}}= & {} \phantom {z} (1 - z) Q^2 ~~ \mathrm {for~ISR}. \end{aligned}$$

Here $Q^2$ is the timelike or spacelike virtuality of the off-shell parton for FSR and ISR, respectively [23]. (For simplicity we only show the formulae in the massless case.) The $p_{\perp \mathrm {evol}}$ would agree with the conventional $p_{\perp }$ of the daughters in a branching if *z* had been defined as the fraction of the lightcone momentum $E + p_{\mathrm {longitudinal}}$. Now it is not, as we shall see, which leads to modest mismatches between $p_{\perp \mathrm {evol}}$ and $p_{\perp }$. In eq. (1) ISR and FSR is actually to be written in terms of its respective $p_{\perp \mathrm {evol}}$, while the MPI $p_{\perp }$ remains the normal one.

**Fig. 3:**
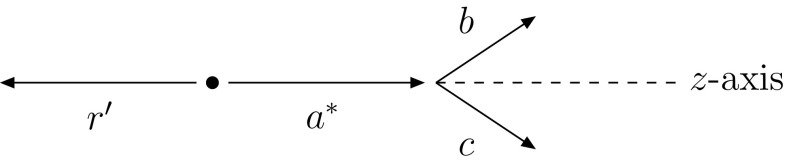
Sketch of the branching $a\rightarrow b + c$ with a recoiler *r*. The *z*-axis is chosen to be along the direction of the momentum of parton *a*

FSR on its own is handled by dipole showering. Each coloured parton *a* is assigned a recoiler *r* that carries the corresponding anticolour in an $N_C \rightarrow \infty$ representation of the colour flow. (Exceptions exist, such as in the decay $\hbox {t} \rightarrow \hbox {bW}^{+}$, where the W is the recoil partner of the $\mathrm {b}$, so as to preserve the t mass.) In a branching $a \rightarrow b + c$ the dipole invariant mass is preserved by the recoiler energy being scaled down, while its direction is maintained. Kinematically, the branching can be split in two steps: $a + r \rightarrow a^* + r' \rightarrow b + c + r'$, where $a^*$ is the intermediate off-shell parton of virtuality $Q^2$ (see Fig. 3). In the first step four-vectors are modified according to

4 $$\begin{aligned} p_{a^*}= & {} p_a + \frac{Q^2}{m_{ar}^2} p_r ~, \end{aligned}$$

5 $$\begin{aligned} p_{r'}= & {} \left( 1 - \frac{Q^2}{m_{ar}^2} \right) p_r. \end{aligned}$$

The *z* definition in the second step is most easily related to the kinematics in the dipole rest frame by $E_b = z E_{a^*}$, $E_c = (1 - z) E_{a^*}$. These $p^2_{\perp \mathrm {evol}}$ and *z* definitions have the advantage that they exactly match on to the singularity structure of MEs, such as the textbook $\gamma ^* / {\mathrm {Z}^0}\rightarrow \mathrm {q}(1) + \,\overline{\mathrm {q}}(2) + \,\mathrm {g}(3)$ one, when radiation from the two dipole ends is combined [32, 35],

6 $$\begin{aligned}&\frac{\mathrm {d}p_{\perp \mathrm {evol},\mathrm {q}}^2}{p_{\perp \mathrm {evol},\mathrm {q}}^2} \, \frac{\mathrm {d}z_{\mathrm {q}}}{1 - z_{\mathrm {q}}} + \frac{\mathrm {d}p_{\perp \mathrm {evol},\overline{\mathrm {q}}}^2}{p_{\perp \mathrm {evol},\overline{\mathrm {q}}}^2} \, \frac{\mathrm {d}z_{\overline{\mathrm {q}}}}{1 - z_{\overline{\mathrm {q}}}} \nonumber \\&\quad = \frac{\mathrm {d}x_1 \, \mathrm {d}x_2}{(1 - x_2) x_3} + \frac{\mathrm {d}x_1 \, \mathrm {d}x_2}{(1 - x_1) x_3} = \frac{\mathrm {d}x_1 \, \mathrm {d}x_2}{(1 - x_1) (1 - x_2)} ~, \end{aligned}$$

with $x_i = 2 E_i/E_{\mathrm {tot}}$. Matrix element corrections therefore are easily implemented (also when generalized to massive kinematics [23, 36]). This would not be the case if the true $p_{\perp }$ had been used instead of $p_{\perp \mathrm {evol}}$ [37], at least with this *z* definition.

ISR on its own is handled with backwards evolution and a global recoil. That is, consider a collision $b + r \rightarrow F$, where *F* may represent a multibody final state. If *b* comes from a previous branching $a \rightarrow b + c$, by backwards evolution, the full process reads $a + r \rightarrow b^* + c + r \rightarrow F' + c$. Note that *r* remains unchanged by the branching in this case. Here $z = m^2_{br} / m^2_{ar}$, which gives a good match to relevant (Mandelstam) ME variables. Considering e.g. emission in a $\mathrm {q}+ \overline{\mathrm {q}}\rightarrow \mathrm {Z}^0$ process, giving $\mathrm {q}+ \overline{\mathrm {q}}\rightarrow \mathrm {Z}^0 + \mathrm {g}$, $\hat{s} = m^2_{\mathrm {Z}} / z$ and

7 $$\begin{aligned} \frac{\mathrm {d}p^2_{\perp \mathrm {evol}}}{p^2_{\perp \mathrm {evol}}} = \frac{\mathrm {d}Q^2}{Q^2} = \frac{\mathrm {d}\hat{t}}{\hat{t}} ~~\mathrm {or}~~ \frac{\mathrm {d}\hat{u}}{\hat{u}}~, \end{aligned}$$

simplifying ME reweighting also here [38]. The $F'$ system is a boosted and rotated copy of *F*, i.e. the internal topology is unchanged. As the backwards evolution continues, the new *F* system also contains the *c* parton of the previous branching.

The ISR and FSR descriptions can be separated so long as colour does not flow between the initial and the final state. Notably, if *F* is a colour singlet state, the ISR approach above is a valid $a + r$ II dipole-language description of the radiation. At hadron colliders this is seldom the case, however, and therefore an FI and IF handling need to be introduced, one way or another, for the colour dipoles stretched between the initial and the final state.

The kinematics of an FI branching gives some differences relative to an FF one. In the dipole rest frame a fraction $Q^2 / m_{ar}^2$ of the recoiler energy is given from the recoiler to the emitter, exactly as in Eq. (4). But the recoiler is not a final-state particle, so the increase of *a* momentum is not compensated anywhere in the final state. Instead the incoming parton that the recoiler represents must have its momentum increased, not decreased, by the same amount as the emitter. That is, its momentum fraction *x* needs to be scaled up as

8 $$\begin{aligned} x_{r'} = \left( 1 + \frac{Q^2}{m_{ar}^2} \right) x_r. \end{aligned}$$

Note that the direction along the incoming beam axis is not affected by this rescaling, and that the kinematics construction therefore inevitably comes to resemble that of Catani–Seymour dipoles [22]. The dipole mass $m_{ar}$ and the squared subcollision mass $\hat{s}$ are increased in the process, the latter by the same factor as $x_r$. As with ISR, the increased *x* value leads to an extra PDF weight

9 $$\begin{aligned} \frac{x_{r'} f_r(x_{r'},p_{\perp }^2)}{x_r f_r(x_r,p_{\perp }^2)} \end{aligned}$$

in the emission probability and Sudakov form factor. This ensures a proper damping of radiation in the $x_{r'} \rightarrow 1$ limit.

So far Pythia has had no implementation of IF dipole ends; all ISR is handled by the II approach. To first approximation this is no problem for the total emission rate, so long as each incoming parton is allowed to radiate according to its full colour charge. In more detail, however, one must beware of a double- or undercounting of the full radiation pattern when it is combined with the FI contribution. Note that this pattern should depend on the scattering angle of the colour flow in a hard process: if colour flows from an incoming parton *i* to a final parton *f* then $m^2_{if} = E_i E_f (1 - \cos \theta _{if})$ sets the phase space available for emission. In [32] an approximate prescription is introduced to dampen FI radiation that otherwise could be doublecounted, but no corresponding procedure is implemented on the ISR side. What is done with ISR, on the other hand, is to implement azimuthal asymmetries in the radiation pattern from colour coherence considerations [39], that lines up radiation off the *i* parton with the azimuthal angle of the *f*, in the same spirit as a dipole would, but presumably not as accurately.

**Fig. 4:**
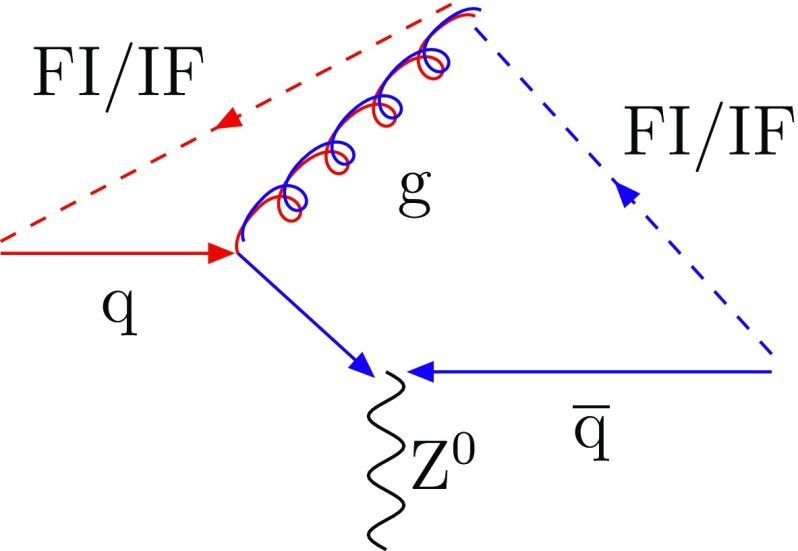
Colour flow for the process $\mathrm {q}+ \overline{\mathrm {q}}\rightarrow \gamma ^* / {\mathrm {Z}^0}+ \mathrm {g}$. The dashed lines represent the colour lines stretching between the dipole ends

While it thus would seem that the dipole $\hbox {IF} + \hbox {FI}$ approach is superior to the global-recoil one, the issue is not always as one-sided. The prime example is $\mathrm {q}+ \overline{\mathrm {q}}\rightarrow \gamma ^* / {\mathrm {Z}^0}$ production. Once a gluon has been emitted from the original $\mathrm {q}+ \overline{\mathrm {q}}$ II dipole, any further emission will be related to the resulting $\mathrm {q}+ \mathrm {g}$ and $\mathrm {g}+ \overline{\mathrm {q}}$ dipoles represented in Fig. 4. Therefore the $\gamma ^* / {\mathrm {Z}^0}$ only receives a recoil in the first step for the dipole approach. With Feynman diagrams, on the other hand, the $\gamma ^* / {\mathrm {Z}^0}$ takes a recoil that is modified as further gluon emissions are considered. In this respect the global-recoil shower strategy is analogous with how resummation techniques [40] are used to sum up the effects of infinitely many gluon emissions on the $p_{\perp }$ spectrum of the $\gamma ^* / {\mathrm {Z}^0}$. This clear defect of the dipole picture has been a main reason to maintain the older global-recoil strategy, with modest improvements.

Nowadays showers are not used on their own when high precision is required, however, but are matched/merged with higher-order MEs [1]. With the kinematics of the hardest four or so emissions based on MEs, and only subsequent ones described by showers, it is reasonable to assume that the $\gamma ^* / {\mathrm {Z}^0}$ $p_{\perp }$ spectrum is not impaired by the lack of further recoils. On a philosophical level, it still reminds us that the dipole picture also is an approximation, and that different approaches should be developed as a means to assess uncertainties, as it has been done e.g. in [41] by using two different recoil strategies.

Finally, it should be mentioned that Pythia also contains a global-recoil option for FSR, not only for ISR. That is, when one final parton radiates, all other final partons are boosted, as a unit, so as to preserve total four-momentum. This option is mainly intended to simplify matching/merging with NLO results, the way they are calculated with the MadGraph5_aMC@NLO program [42]. Typically global recoil is therefore only used in the first one or two branchings, whereafter one switches to the dipole picture. A similar strategy could be envisioned for ISR, even if it has not been studied here.

## The new approach

### Kinematics for IF emissions

**Fig. 5:**
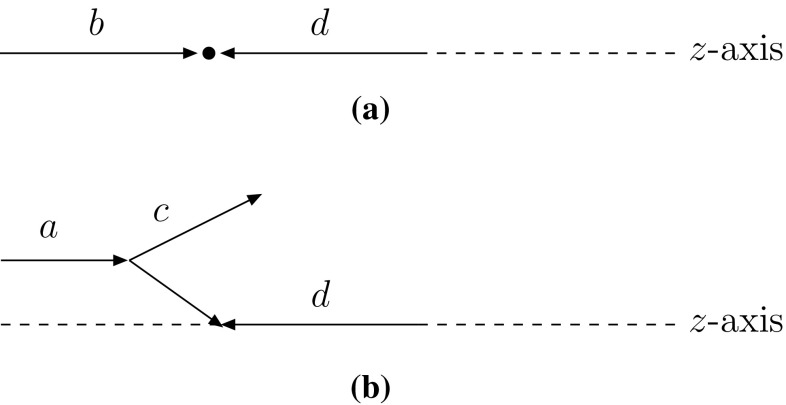
ISR kinematics. **a** Before branching: partons *b* and *d* incoming in the $\pm \, z$ direction. **b** After the branching $a \rightarrow b + c$, now with *a* and *d* are along the *z*-axis

Let us consider a collision in the event frame between two incoming partons *b* and *d* with four-momenta $p_{b,d} = x_{b,d}(\sqrt{s}/2)(1;0,0,\pm \, 1)$, where $\sqrt{s}$ is the total centre-of-mass energy and $x_{b,d}$ are the four-momentum fractions. The two partons are taken as massless. A sketch of the process is given in Fig. 5a.

When evolving backwards in time, the parton *b* is seen as coming from the branching $a \rightarrow b + c$. The parton *b* hence obtains a spacelike virtuality $Q^2 > 0$, and the previously established kinematics has to be modified. It is now the parton *a* which should be the incoming one, with four-momentum $p'_a = x_a(\sqrt{s}/2)(1;0,0,1)$ in the event frame, Fig. 5b (primed labels denote momenta after the branching has been considered). The parton *d* keeps its original four-momentum, so $p'_d = p_d$. The variable *z* is defined as $z = x_b/x_a$ or, in terms of invariant masses, as $z = m_{bd}^2/m_{ad}^2$. This holds since *a*, *b* and *d* are always taken as massless, so that $m_{bd}^2 = (p_b + p_d)^2 = x_b\,x_d\,s$ and $m_{ad}^2 = (p'_a + p'_d)^2 = x_a\,x_d\,s$ [23]. Therefore also $p'_a = p_b / z$.

In the default global-recoil approach, the whole final state created by $\{b + d\}$ obtains changed momenta. In the new scheme, the recoil is instead taken by the single final parton *f*, which is the one colour-connected to parton *b*. In the following, this parton *f* is referred to as the colour partner.

Before the branching, four-momentum conservation gives

10 $$\begin{aligned} p_b + p_d = p_f + p_F, \end{aligned}$$

where *F* represents the system of all final partons except for the colour partner. After the branching $a \rightarrow b + c$ instead

11 $$\begin{aligned} p'_a + p'_d = p'_f + p'_F + p'_c. \end{aligned}$$

The local recoil ansatz implies that $p'_d = p_d$ and $p'_F = p_F$, while $p'_f \ne p_f$. The difference between the above two equations gives

12 $$\begin{aligned} p'_a - p_b = p'_f - p_f + p'_c, \end{aligned}$$

where $p_b$, $p'_a$ and $p_f$ are known. Together $p'_f$ and $p'_c$ contain eight unknowns. Equation (12) gives four constraints, and three others are

13 $$\begin{aligned} p_c'^2 = m_c^2, \quad p_f'^2 = p_f^2 = m_f^2, p_b'^2 = (p'_a - p'_c)^2 = -Q^2.\nonumber \\ \end{aligned}$$

The remaining degree of freedom is the azimuthal angle $\varphi$ of the emitted parton *c*, which can be generated isotropically in the dipole rest frame. This is one of the advantages of the new approach: azimuthal asymmetries due to colour coherence effects are automatically generated when the system is boosted and rotated back to the event rest frame.

Given the $Q^2$ and *z* variables, the unknown four-momenta can be expressed in the $\{b + f\}$ rest frame, here denoted as $\hat{p}$. Before the branching

14 $$\begin{aligned} \hat{p}_b= & {} \left( \frac{m_{\mathrm {dip}}^2 - m_f^2}{2m_{\mathrm {dip}}};0,0, \frac{m_{\mathrm {dip}}^2 - m_f^2}{2m_{\mathrm {dip}}}\right) , \end{aligned}$$

15 $$\begin{aligned} \hat{p}_f= & {} \left( \frac{m_{\mathrm {dip}}^2 + m_f^2}{2m_{\mathrm {dip}}};0,0, - \frac{m_{\mathrm {dip}}^2 - m_f^2}{2m_{\mathrm {dip}}}\right) , \end{aligned}$$

with $m_{\mathrm {dip}}^2 = (p_b + p_f)^2$. Note that the dipole mass is not conserved during the branching. After the branching

16 $$\begin{aligned} \hat{p}'_c= & {} \left( \frac{1}{z} - 1 \right) \hat{p}_b + \hat{p}_{\mathrm {shift}}, \end{aligned}$$

17 $$\begin{aligned} \hat{p}'_f= & {} \hat{p}_f - \hat{p}_{\mathrm {shift}}, \end{aligned}$$

with

18 $$\begin{aligned} \hat{p}_{\mathrm {shift}}&= \left( \phantom {\left. - \frac{Q^2}{2m_{\mathrm {dip}}} - z\frac{m_f^2}{m_{\mathrm {dip}}} \frac{Q^2 + m_c^2}{m_{\mathrm {dip}}^2 - m_f^2} \right) } \frac{(2z - 1)Q^2}{2m_{\mathrm {dip}}} + z\frac{m_c^2}{m_{\mathrm {dip}}}; \hat{p}_{\perp } \cos \varphi , \hat{p}_{\perp } \sin \varphi , \right. \nonumber \\&\quad \left. - \frac{Q^2}{2m_{\mathrm {dip}}} - z\frac{m_f^2}{m_{\mathrm {dip}}} \frac{Q^2 + m_c^2}{m_{\mathrm {dip}}^2 - m_f^2} \right) ~, \end{aligned}$$

where $\hat{p}_{\perp }$ is the transverse momentum of parton *c* with respect to the dipole axis

19 $$\begin{aligned} \hat{p}_{\perp }^2= & {} \left( (1 - z)(Q^2 + m_c^2) - m_c^2\right) \left( 1 - z\frac{Q^2 + m_c^2}{m_{\mathrm {dip}}^2 - m_f^2}\right) \nonumber \\&-\, m_f^2\left( z\frac{Q^2 + m_c^2}{m_{\mathrm {dip}}^2 - m_f^2}\right) ^2. \end{aligned}$$

The same set of rotations and boosts as used to get to the $\{b + f\}$ rest frame can then be inverted to bring $\hat{p}'_c$ and $\hat{p}'_f$ back to $p'_c$ and $p'_f$ in the event rest frame.

### Gluon emission in DIS

Now that the kinematics has been set up, the emission pattern of IF systems can be analyzed, as described by $\mathrm {d}\mathscr {P}_{\mathrm {ISR}}$ in Eq. (1), using standard DGLAP splitting kernels and backwards evolution as for II dipoles [23]. For simplicity the PDF corrections, cf. Eq. (9), are omitted in the following discussion.

**Fig. 6:**
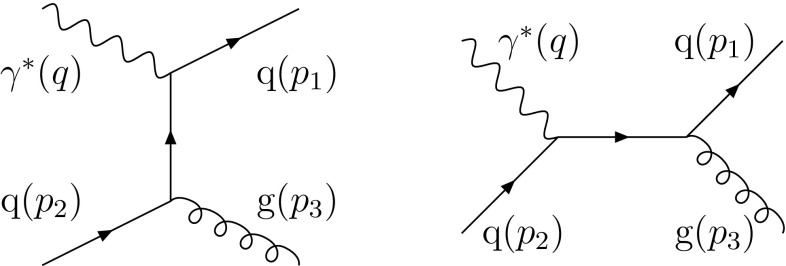
The two Feynman graphs contributing to the process $\gamma ^* + \mathrm {q}\rightarrow \mathrm {q}+ \mathrm {g}$ at $\mathscr {O}(\alpha _{\mathrm {em}}\alpha _{\mathrm {s}})$. The assigned four-momenta of the particles are given in brackets

As already explained, the prime example is gluon emission in DIS, where a single FI/IF dipole naturally appears. At $\mathscr {O}(\alpha _{\mathrm {em}}\alpha _{\mathrm {s}})$ two Feynman graphs lead to this process, Fig. 6.

From a parton-shower point of view, the final state $\mathrm {q}+ \mathrm {g}$ can be generated either via gluon emission off the IF system or off the FI one. The standard FSR machinery implemented in the existing Pythia already includes FI systems. The aim now is to calculate the contribution of an IF emission in this case and combine it with the contribution from the FI emission in order to compare with the full matrix element, which also includes interference effects. This is done by using the IF kinematics previously established.

To study this kind of processes, the $(x,z_1)$ variables used by Catani and Seymour [22] are convenient:

20 $$\begin{aligned} x = \frac{|q^2|}{2\,p_2\cdot q}, \quad z_1 = \frac{p_{1}\cdot p_2}{p_2\cdot q}. \end{aligned}$$

The usual $Q^2$ and *z* variables can be expressed in terms of $(x,z_1)$, noting that $\hat{p}'_a = p_2$, $\hat{p}'_f = p_{1}$, $\hat{p}'_c = p_{3}$ and $q = p_{1} + p_{3} - p_2$. Setting $m_f = m_c = 0$ for simplicity, one finds that

21 $$\begin{aligned} z = x, \quad Q^2 = m_{\mathrm {dip}}^2\frac{1 - z_1}{x}, \quad \mathrm {d}Q^2 \, \mathrm {d}z = \frac{m_{\mathrm {dip}}^2}{x} \, \mathrm {d}x \, \mathrm {d}z_1. \end{aligned}$$

Therefore the probability for an IF-type branching $\mathrm {q}\rightarrow \mathrm {q}+ \mathrm {g}$ is

22 $$\begin{aligned} \mathrm {d}\mathscr {P}_{\mathrm {q}\rightarrow \mathrm {q}\mathrm {g}}^{\mathrm {IF}}&= \frac{4}{3} \, \frac{\alpha _{\mathrm {s}}}{2\pi } \, \frac{\mathrm {d}Q^2}{Q^2} \, \frac{1 + z^2}{1 - z} \, \mathrm {d}z \nonumber \\&\quad = \frac{4}{3}\,\frac{\alpha _{\mathrm {s}}}{2\pi }\, \frac{(1 + x^2)}{(1 - x)(1 - z_1)} \,\mathrm {d}x\,\mathrm {d}z_1 ~. \end{aligned}$$

The soft ($z = 1$) and collinear ($Q^2 = 0$) singularities are mapped onto the singularities $x = 1$ and $z_1 = 1$, respectively. This is a striking result, since the full matrix-element expression for $\gamma ^* + \mathrm {q}\rightarrow \mathrm {q}+ \mathrm {g}$ has exactly the same two singularities. Overall, only the numerator is slightly different, $z_1^2 + x^2$ [22] instead of our $1 + x^2$ . (There are also finite terms that we leave aside here.) That is, the IF-type branching $\mathrm {q}\rightarrow \mathrm {q}+ \mathrm {g}$ generates both singularities of the full cross-section on its own.

The recoil of an FF dipole emission is not uniquely specified [12]. In Pythia the FF dipole is artificially split into two dipole ends according to Eq. (6), which allows to have two different phase-space mappings, where the recoiler in each case does not change its direction of motion. For the FI/IF dipole there is no such freedom: the two incoming partons must always be parallel with the beam axis, whether an emission is viewed as an FI or an IF one [23, 37]. Also, the momentum fraction of the initial-state dipole end has to be increased after the branching, cf. Eq. (8), in order to absorb the virtuality. Therefore there will only be one phase-space mapping. The full emission rate could still be viewed as a sum of one IF and one FI contribution, by splitting the expression in Eq. (22) in the spirit of Eq. (6). A corresponding reweighting of the IF rate would be easily achieved. Unfortunately the $(Q^2,z)$ variables of an FI dipole-end emission are not trivially related to the $(x,z_1)$ ones. This could be overcome by a reweighting with the appropriate Jacobian, but would be more cumbersome and not bring any benefits relative to using only $\mathrm {d}\mathscr {P}_{\mathrm {q}\rightarrow \mathrm {q}\mathrm {g}}^{\mathrm {IF}}$, which on its own reproduces the full answer so well. In the end, working only with IF emissions then seems reasonable.

### Generalisation

The previous example was for the branching $\mathrm {q}\rightarrow \mathrm {q}+ \mathrm {g}$. It is now important to verify whether these features, which appear for this specific branching, are also present for the other kinds of branchings. Therefore, the emission probabilities for IF systems and FI systems will be compared. The objective is to check whether the emission pattern of the FI type can be described by the IF type only, at least as far as the singularity structure goes. Invariant masses will be used as variables to make the comparison easier between ISR and FSR. For an IF branching $a \rightarrow b + c$ they are $m_{ac}^2 = (p'_a + p'_c)^2$ and $m_{fc}^2 = (p'_f + p'_c)^2$. In the FI case we have $m_{bc}^2 = (p'_b + p'_c)^2$ and $m_{rc}^2 = (p'_{r} + p'_c)^2$, where *r* is the recoiling colour partner in the initial state (recall Eq. (8) and Fig. 3). For massless partons this gives

23 $$\begin{aligned} z = \frac{m_{\mathrm {dip}}^2}{m_{\mathrm {dip}}^2 + m_{fc}^2}, \quad Q^2 = m_{ac}^2 \end{aligned}$$

for IF, and

24 $$\begin{aligned} z = \frac{m_{\mathrm {dip}}^2(m_{\mathrm {dip}}^2 + m_{bc}^2) - m_{rc}^2(m_{\mathrm {dip}}^2 - m_{bc}^2)}{(m_{\mathrm {dip}}^2 + m_{bc}^2)^2}, \quad Q^2 = m_{bc}^2 \end{aligned}$$

for FI. The limits $m_{ac}^2\rightarrow 0$ and $m_{rc}^2\rightarrow 0$ can be associated with IF emissions, and the limits $m_{bc}^2\rightarrow 0$ and $m_{fc}^2\rightarrow 0$ with FI ones. Table 1 summarizes the singularity structure of the branching probabilities for IF and FI.

**Table 1:** Singularity structure of the probability of emission for IF and FI. For IF, the parton *b* in the branching $a\rightarrow b + c$ is the one which was incoming before the backward evolution. The mapping between IF and FI labels is the following: $a\leftrightarrow r$, $f\leftrightarrow b$ and $c\leftrightarrow c$

Branching $a\rightarrow bc$	Singularities of $P_{a\rightarrow bc}(z)$	Singularities of $\mathrm {d}\mathscr {P}_{a\rightarrow bc}^{\mathrm {IF}}$	Singularities of $\mathrm {d}\mathscr {P}_{a\rightarrow bc}^{\mathrm {FI}}$
$\mathrm {q}\rightarrow \mathrm {q}\mathrm {g}$	$\displaystyle \frac{1}{1 - z}$	$\displaystyle \frac{\mathrm {d}m_{ac}^2\,\mathrm {d}m_{fc}^2}{m_{ac}^2\,m_{fc}^2}$	$\displaystyle \frac{\mathrm {d}m_{bc}^2\,\mathrm {d}m_{rc}^2}{m_{bc}^2}$
$\mathrm {q}\rightarrow \mathrm {g}\mathrm {q}$	$\displaystyle \frac{1}{z}$	$\displaystyle \frac{\mathrm {d}m_{ac}^2\,\mathrm {d}m_{fc}^2}{m_{ac}^2}$	$\displaystyle \frac{\mathrm {d}m_{bc}^2\,\mathrm {d}m_{rc}^2}{m_{bc}^2}$
$\mathrm {g}\rightarrow \mathrm {g}\mathrm {g}$	$\displaystyle \frac{1}{z(1 - z)}$	$\displaystyle \frac{\mathrm {d}m_{ac}^2\,\mathrm {d}m_{fc}^2}{m_{ac}^2\,m_{fc}^2}$	$\displaystyle \frac{\mathrm {d}m_{bc}^2\,\mathrm {d}m_{rc}^2}{m_{bc}^2}$
$\mathrm {g}\rightarrow \mathrm {q}\overline{\mathrm {q}}$	1	$\displaystyle \frac{\mathrm {d}m_{ac}^2\,\mathrm {d}m_{fc}^2}{m_{ac}^2}$	$\displaystyle \frac{\mathrm {d}m_{bc}^2\,\mathrm {d}m_{rc}^2}{m_{bc}^2}$

For the branchings $\mathrm {q}\rightarrow \mathrm {q}\mathrm {g}$ and $\mathrm {g}\rightarrow \mathrm {g}\mathrm {g}$, $\mathrm {d}\mathscr {P}_{a\rightarrow bc}^{\mathrm {IF}}$ contains both the singularities $m_{ac}^2 = 0$ and $m_{fc}^2 = 0$. The first one is expected since it is an IF system, but the singularity $m_{fc}^2 = 0$ is actually the same as the singularity $m_{bc}^2 = 0$ which shows up in $\mathrm {d}\mathscr {P}_{a\rightarrow bc}^{\mathrm {FI}}$. Therefore, by analogy with the DIS case, the probability $\mathrm {d}\mathscr {P}_{a\rightarrow bc}^{\mathrm {IF}}$ seems sufficient to describe the emission pattern of both IF and FI systems in those cases. For the branchings $\mathrm {q}\rightarrow \mathrm {g}\mathrm {q}$ and $\mathrm {g}\rightarrow \mathrm {q}\overline{\mathrm {q}}$, on the other hand, $\mathrm {d}\mathscr {P}_{a\rightarrow bc}^{\mathrm {IF}}$ does not obtain any additional singularity that could be associated with FI emissions. Here the flavour configurations are also separate, see further below, so IF and FI anyway have to be considered separately. All possible flavour configurations have been studied, see Table 2.

**Table 2:** The four configurations of an original FI/IF dipole, with all the branchings that can occur for it. The probability of emission which is used to describe the branching has been specified

Dipole configuration: initial − final ends	Branching $a\rightarrow bc$	Emission pattern described with:
$\mathrm {q}_{\mathrm {i}} - \mathrm {q}_{\mathrm {f}}$	$\mathrm {q}_{\mathrm {f}}\rightarrow \mathrm {q}\mathrm {g}$	$\mathrm {d}\mathscr {P}_{\mathrm {q}\rightarrow \mathrm {q}_{\mathrm {i}}\mathrm {g}}^{\mathrm {IF}}$
	$\mathrm {q}\rightarrow \mathrm {q}_{\mathrm {i}} \mathrm {g}$	$\mathrm {d}\mathscr {P}_{\mathrm {q}\rightarrow \mathrm {q}_{\mathrm {i}}\mathrm {g}}^{\mathrm {IF}}$
	$\mathrm {g}\rightarrow \mathrm {q}_{\mathrm {i}} \overline{\mathrm {q}}$	$\mathrm {d}\mathscr {P}_{\mathrm {g}\rightarrow \mathrm {q}_{\mathrm {i}}\overline{\mathrm {q}}}^{\mathrm {IF}}$
$\mathrm {g}_{\mathrm {i}} - \mathrm {g}_{\mathrm {f}}$	$\mathrm {g}_{\mathrm {f}} \rightarrow \mathrm {g}\mathrm {g}$	$\mathrm {d}\mathscr {P}_{\mathrm {g}\rightarrow \mathrm {g}_{\mathrm {i}} \mathrm {g}}^{\mathrm {IF}}$
	$\mathrm {g}\rightarrow \mathrm {g}_{\mathrm {i}}\mathrm {g}$	$\mathrm {d}\mathscr {P}_{\mathrm {g}\rightarrow \mathrm {g}_{\mathrm {i}}\mathrm {g}}^{\mathrm {IF}}$
	$\mathrm {q}\rightarrow \mathrm {g}_{\mathrm {i}} \mathrm {q}$	$\mathrm {d}\mathscr {P}_{\mathrm {q}\rightarrow \mathrm {g}_{\mathrm {i}} \mathrm {q}}^{\mathrm {IF}}$
	$\mathrm {g}_{\mathrm {f}}\rightarrow \mathrm {q}\overline{\mathrm {q}}$	$\mathrm {d}\mathscr {P}_{\mathrm {g}_{\mathrm {f}} \rightarrow \mathrm {q}\overline{\mathrm {q}}}^{\mathrm {FI}}$
$\mathrm {q}_{\mathrm {i}} - \mathrm {g}_{\mathrm {f}}$	$\mathrm {g}_{\mathrm {f}} \rightarrow \mathrm {g}\mathrm {g}$	$\mathrm {d}\mathscr {P}_{\mathrm {q}\rightarrow \mathrm {q}_{\mathrm {i}}\mathrm {g}}^{\mathrm {IF}}$
	$\mathrm {q}\rightarrow \mathrm {q}_{\mathrm {i}} \mathrm {g}$	$\mathrm {d}\mathscr {P}_{\mathrm {q}\rightarrow \mathrm {q}_{\mathrm {i}}\mathrm {g}}^{\mathrm {IF}}$
	$\mathrm {g}\rightarrow \mathrm {q}_{\mathrm {i}} \overline{\mathrm {q}}$	$\mathrm {d}\mathscr {P}_{\mathrm {g}\rightarrow \mathrm {q}_{\mathrm {i}}\overline{\mathrm {q}}}^{\mathrm {IF}}$
	$\mathrm {g}_{\mathrm {f}}\rightarrow \mathrm {q}\overline{\mathrm {q}}$	$\mathrm {d}\mathscr {P}_{\mathrm {g}_{\mathrm {f}} \rightarrow \mathrm {q}\overline{\mathrm {q}}}^{\mathrm {FI}}$
$\mathrm {g}_{\mathrm {i}} - \mathrm {q}_{\mathrm {f}}$	$\mathrm {q}_{\mathrm {f}} \rightarrow \mathrm {q}\mathrm {g}$	$\mathrm {d}\mathscr {P}_{\mathrm {g}\rightarrow \mathrm {g}_{\mathrm {i}}\mathrm {g}}^{\mathrm {IF}}$
	$\mathrm {g}\rightarrow \mathrm {g}_{\mathrm {i}}\mathrm {g}$	$\mathrm {d}\mathscr {P}_{\mathrm {g}\rightarrow \mathrm {g}_{\mathrm {i}}\mathrm {g}}^{\mathrm {IF}}$
	$\mathrm {q}\rightarrow \mathrm {g}_{\mathrm {i}} \mathrm {q}$	$\mathrm {d}\mathscr {P}_{\mathrm {q}\rightarrow \mathrm {g}_{\mathrm {i}}\mathrm {q}}^{\mathrm {IF}}$

The general strategy is to use as much as possible the branching probabilities of the IF type. Take the example of $\mathrm {q}_{\mathrm {i}} - \mathrm {g}_{\mathrm {f}}$. A gluon emission might either come from the ISR $\mathrm {q}\rightarrow \mathrm {q}_{\mathrm {i}} \mathrm {g}$ or from the FSR $\mathrm {g}_{\mathrm {f}}\rightarrow \mathrm {g}\mathrm {g}$. The same final configuration is obtained in both cases. As for the DIS case, the double-singularity structure of $\mathrm {d}\mathscr {P}_{\mathrm {q}\rightarrow \mathrm {q}_{\mathrm {i}}\mathrm {g}}^{\mathrm {IF}}$ can be used to describe both the ISR and the FSR, with a smooth transition between the two. The only problem is a slight mismatch in colour factors between $\mathrm {q}\rightarrow \mathrm {q}_{\mathrm {i}} \mathrm {g}$ and $\mathrm {g}_{\mathrm {f}}\rightarrow \mathrm {g}\mathrm {g}$, which will be addressed in the next section.

Now instead consider the ISR branching $\mathrm {g}\rightarrow \mathrm {q}_{\mathrm {i}}\overline{\mathrm {q}}$ off the same original $\mathrm {q}_{\mathrm {i}} - \mathrm {g}_{\mathrm {f}}$ dipole. This leads to a final flavour configuration that cannot be obtained by FSR off $\mathrm {g}_{\mathrm {f}}$. The emission pattern is then described with $\mathrm {d}\mathscr {P}_{\mathrm {g}\rightarrow \mathrm {q}_{\mathrm {i}}\overline{\mathrm {q}}}^{\mathrm {IF}}$, which has only one singularity, as wanted. The converse applies for the FSR $\mathrm {g}_{\mathrm {f}}\rightarrow \mathrm {q}\overline{\mathrm {q}}$, which can only be described by $\mathrm {d}\mathscr {P}_{\mathrm {g}_{\mathrm {f}}\rightarrow \mathrm {q}\overline{\mathrm {q}}}^{\mathrm {FI}}$ since there is no ISR which would give an equivalent final configuration.

In summary we see that the dipole picture works elegantly for the emission of gluons, but is less elegant when the quark flavour content is changed, a well-known observation since long [43].

### Some technical aspects

Some technical issues are addressed in this section. They relate to the way the basic ideas are implemented in Pythia. These aspects are important, but not essential to understand the main ideas of this article.

#### Phase-space cuts

The kinematics for an IF emission has been derived in Sect. 3.1. Also the allowed $(p^2_{\perp \mathrm {evol}}, z)$ phase-space region has to be known. Firstly, a lower cutoff $p^2_{\perp \mathrm {evol}}> p_{\perp \mathrm {cutoff}}^2$ is imposed, where $p_{\perp \mathrm {cutoff}}\approx 1~\hbox {GeV}$ represents a scale where perturbation theory breaks down and confinement takes over. (Actually, a smooth damping of perturbative emissions is used rather than a sharp cutoff.) The range $[z_{\mathrm {min}},z_{\mathrm {max}}]$ of allowed *z* values is obtained from the physical condition $\hat{p}_{\perp }^2 > 0$ [37]. To this end Eq. (19) is rewritten in terms of the evolution variable $p^2_{\perp \mathrm {evol}}$. For a massless emitted parton ($m_c = 0$) the evolution variable is $p^2_{\perp \mathrm {evol}}= (1 - z) Q^2$ and

25 $$\begin{aligned} \hat{p}_{\perp }^2 = p^2_{\perp \mathrm {evol}}\left( 1 - \frac{z}{1 - z} \, \frac{p^2_{\perp \mathrm {evol}}}{m_{\mathrm {red}}^2} \right) - m_f^2 \left( \frac{z}{1 - z} \, \frac{p^2_{\perp \mathrm {evol}}}{m_{\mathrm {red}}^2} \right) ^2. \end{aligned}$$

with $m_{\mathrm {red}}^2 = m_{\mathrm {dip}}^2 - m_f^2$. The constraint $\hat{p}_{\perp }^2 > 0$ then gives

26 $$\begin{aligned} z_{\mathrm {max}}(p^2_{\perp \mathrm {evol}}) = \frac{2 + \left( p^2_{\perp \mathrm {evol}}- p_{\perp \mathrm {evol}}\sqrt{p^2_{\perp \mathrm {evol}}+ 4m_f^2}\right) / m_{\mathrm {red}}^2}{2\left( 1 + \frac{p^2_{\perp \mathrm {evol}}}{m_{\mathrm {red}}^2} - \frac{m_f^2}{m_{\mathrm {red}}^2} \frac{p^2_{\perp \mathrm {evol}}}{m_{\mathrm {red}}^2}\right) }.\nonumber \\ \end{aligned}$$

An overestimate independent of $p^2_{\perp \mathrm {evol}}$ is required in the veto algorithm used for the downwards evolution in $p^2_{\perp \mathrm {evol}}$ [24]. Since $z_{\mathrm {max}}$ is strictly decreasing with $p^2_{\perp \mathrm {evol}}$ the value at $p_{\perp \mathrm {cutoff}}^2$ can be used to this end. The lower limit comes from $x_a = x_b / z \le 1$, which implies $z \ge x_b$.

The range previously found is valid for a massless emitted parton. The case where $m_c\ne 0$ occurs e.g. for $\mathrm {g}\rightarrow \mathrm {Q}\overline{\mathrm {Q}}$, with $\mathrm {Q} = \mathrm {c},\mathrm {b}$. The procedure is the same as above, but now $p^2_{\perp \mathrm {evol}}= (1 - z)(Q^2 + m_c^2)$ and

27 $$\begin{aligned} \hat{p}_{\perp }^2&= (p^2_{\perp \mathrm {evol}}- m_c^2) \left( 1 - \frac{z}{1 - z}\, \frac{p^2_{\perp \mathrm {evol}}}{m_{\mathrm {red}}^2} \right) \nonumber \\&\quad - m_f^2 \left( \frac{z}{1 - z}\,\frac{p^2_{\perp \mathrm {evol}}}{m_{\mathrm {red}}^2}\right) ^2, \end{aligned}$$

which gives

28 $$\begin{aligned} z_{\mathrm {max}}(p^2_{\perp \mathrm {evol}})= & {} \frac{p^2_{\perp \mathrm {evol}}- m_c^2 + \frac{p^2_{\perp \mathrm {evol}}}{2m_{\mathrm {red}}^2} (p^2_{\perp \mathrm {evol}}- m_c^2 - p_{\perp \mathrm {temp}}^2)}{p^2_{\perp \mathrm {evol}}- m_c^2 + \frac{p^2_{\perp \mathrm {evol}}(p^2_{\perp \mathrm {evol}}- m_c^2)}{m_{\mathrm {red}}^2} - m_f^2 \frac{p_{\perp \mathrm {evol}}^4}{m_{\mathrm {red}}^4}}~, \nonumber \\ p_{\perp \mathrm {temp}}^2= & {} \sqrt{(p^2_{\perp \mathrm {evol}}- m_c^2)^2 + 4m_f^2(p^2_{\perp \mathrm {evol}}- m_c^2)}.\nonumber \\ \end{aligned}$$

This expression is rather cumbersome. If the colour partner is a gluon or a light quark, $m_f = 0$, however, it simplifies to

29 $$\begin{aligned} z_{\mathrm {max}}(p^2_{\perp \mathrm {evol}})&= \frac{p^2_{\perp \mathrm {evol}}- m_c^2}{p^2_{\perp \mathrm {evol}}- m_c^2 + \frac{p^2_{\perp \mathrm {evol}}(p^2_{\perp \mathrm {evol}}- m_c^2)}{m_{\mathrm {red}}^2}} \nonumber \\&= \frac{m_{\mathrm {red}}^2}{m_{\mathrm {red}}^2 + p^2_{\perp \mathrm {evol}}}. \end{aligned}$$

The $z_{\mathrm {max}}$ function is strictly decreasing in that specific case, and can be overestimated by $\tilde{z}_{\mathrm {max}} = z_{\mathrm {max}}(m_c^2)$, since the evolution is such that $p^2_{\perp \mathrm {evol}}\ge m_c^2$ in the massive case.

For $m_f\ne 0$ the function $z_{\mathrm {max}}$ is not strictly decreasing anymore. It is bounded from above by the function for the $m_f = 0$ case, however. Therefore the overestimate $\tilde{z}_{\mathrm {max}} = m_{\mathrm {red}}^2/(m_{\mathrm {red}}^2 + m_c^2)$ can be used also for $m_f > 0$. The lower limit remains $\tilde{z}_{\mathrm {min}} = x_b$.

#### Colour factors

When a dipole is stretched between a quark and a gluon the two radiate with different colour factors, $C_F = 4/3$ for the former and $C_A/ 2 = 3/2$ for the latter, where the 1 / 2 for the gluon comes from its radiation being split between two dipoles. More precisely, one can write the $\mathrm {g}\rightarrow \mathrm {g}\mathrm {g}$ splitting kernel as [12]:

30 $$\begin{aligned} \begin{aligned} P_{\mathrm {g}\rightarrow \mathrm {g}\mathrm {g}}(z)&= C_A\,\frac{(1-z(1-z))^2}{z(1-z)} \\&= \frac{C_A}{2}\left( \frac{1+z^3}{1-z} + \frac{1+(1-z)^3}{z} \right) = C_A\,\frac{1+z^3}{1-z} , \end{aligned} \end{aligned}$$

where the last equality is by relabelling symmetry of the two gluons. In the dipole approach, the differences between a $\mathrm {q}\rightarrow \mathrm {q}\mathrm {g}$ and a $\mathrm {g}\rightarrow \mathrm {g}\mathrm {g}$ branching thus are the colour factors, $C_F$ vs. $C_A/2$, and the numerators of the splitting kernels, $1 + z^2$ vs. $1 + z^3$. In the soft-gluon limit, $z \rightarrow 1$, only the former difference survives.

For a $\mathrm {q}_{\mathrm {i}} - \mathrm {g}_{\mathrm {f}}$ dipole, a description purely in terms of IF radiation therefore will underestimate the $\mathrm {g}_{\mathrm {f}}\rightarrow \mathrm {g}\mathrm {g}$ rate by a factor $2 C_F / C_A = 8 / 9$. The idea is to find a compensating smooth weight, which is unity for a gluon emission off $\mathrm {q}_{\mathrm {i}}$ and $C_A/(2C_F) = 9/8$ for one off $\mathrm {g}_{\mathrm {f}}$. Using $1/m^2$ as a measure of proximity, we have chosen the weight

31 $$\begin{aligned} w_{\mathrm {q}_{\mathrm {i}} - \mathrm {g}_{\mathrm {f}}} = \frac{m_{fc}^2 + \frac{C_A}{2C_F}\,m_{ac}^2}{m_{fc}^2 + m_{ac}^2}. \end{aligned}$$

With this choice, $w_{\mathrm {q}_{\mathrm {i}} - \mathrm {g}_{\mathrm {f}}}\rightarrow 1$ for $m_{ac}^2\rightarrow 0$ (emission from $\mathrm {q}_{\mathrm {i}}$) and $w_{\mathrm {q}_{\mathrm {i}} - \mathrm {g}_{\mathrm {f}}}\rightarrow C_A/(2C_F)$ for $m_{fc}^2\rightarrow 0$ (emission from $\mathrm {g}_{\mathrm {f}}$).

Let us now see in more detail how the IF branching probability $w_{\mathrm {q}_{\mathrm {i}} - \mathrm {g}_{\mathrm {f}}}\,\mathrm {d}\mathscr {P}_{\mathrm {q}\rightarrow \mathrm {q}_{\mathrm {i}}\mathrm {g}}^{\mathrm {IF}}$ leads to the gluon-radiation pattern of the full $\mathrm {q}_{\mathrm {i}} - \mathrm {g}_{\mathrm {f}}$ dipole on its own. The IF kinematics (23) leads to the following Jacobian

32 $$\begin{aligned} \mathrm {d}Q^2\,\mathrm {d}z = \frac{m_{\mathrm {dip}}^2}{(m_{\mathrm {dip}}^2+m_{fc}^2)^2}\mathrm {d}m_{fc}^2\,\mathrm {d}m_{ac}^2. \end{aligned}$$

Therefore, the branching probability can be written as

33 $$\begin{aligned} w_{\mathrm {q}_{\mathrm {i}} - \mathrm {g}_{\mathrm {f}}}\,\mathrm {d}\mathscr {P}_{\mathrm {q}\rightarrow \mathrm {q}_\mathrm {i} \mathrm {g}}^{\mathrm {IF}}&=w_{\mathrm {q}_{\mathrm {i}} - \mathrm {g}_{\mathrm {f}}}\,C_F \, \frac{\alpha _{\mathrm {s}}}{2\pi } \, \frac{\mathrm {d}Q^2}{Q^2} \,\frac{1 + z^2}{1 - z} \, \mathrm {d}z \nonumber \\&= \frac{m_{fc}^2 + \frac{C_A}{2C_F}\,m_{ac}^2}{m_{fc}^2 + m_{ac}^2}\,C_F \, \frac{\alpha _{\mathrm {s}}}{2\pi }\,\frac{\mathrm {d}m_{ac}^2}{m_{ac}^2}\,\frac{\mathrm {d}m_{fc}^2}{m_{fc}^2} \nonumber \\&\quad \times \left( 1+\left( \frac{m_{\mathrm {dip}}^2}{m_{\mathrm {dip}}^2+m_{fc}^2}\right) ^2\right) \,\frac{m_{\mathrm {dip}}^2}{m_{\mathrm {dip}}^2+m_{fc}^2}. \end{aligned}$$

When $m_{ac}^2 \rightarrow 0$, the emission can be associated to $\mathrm {q}_\mathrm {i}$ and one gets

34 $$\begin{aligned} \begin{aligned} w_{\mathrm {q}_{\mathrm {i}} - \mathrm {g}_{\mathrm {f}}}\,\mathrm {d}\mathscr {P}_{\mathrm {q}\rightarrow \mathrm {q}_\mathrm {i} \mathrm {g}}^{\mathrm {IF}}&\sim C_F \, \frac{\alpha _{\mathrm {s}}}{2\pi }\,\frac{\mathrm {d}m_{ac}^2}{m_{ac}^2}\,\frac{\mathrm {d}m_{fc}^2}{m_{fc}^2} \\&\times \left( 1+\left( \frac{m_{\mathrm {dip}}^2}{m_{\mathrm {dip}}^2+m_{fc}^2}\right) ^2\right) \,\frac{m_{\mathrm {dip}}^2}{m_{\mathrm {dip}}^2+m_{fc}^2}, \end{aligned} \end{aligned}$$

which leads to the right colour factor $C_F$ with the right singularity structure. For $m_{fc}^2 \rightarrow 0$, the emission is seen as coming from $\mathrm {g}_\mathrm {f}$ and equation (33) gives

35 $$\begin{aligned} w_{\mathrm {q}_{\mathrm {i}} - \mathrm {g}_{\mathrm {f}}}\,\mathrm {d}\mathscr {P}_{\mathrm {q}\rightarrow \mathrm {q}_\mathrm {i} \mathrm {g}}^{\mathrm {IF}}\sim \frac{C_A}{2}\,\frac{\alpha _{\mathrm {s}}}{2\pi }\,\frac{\mathrm {d}m_{ac}^2}{m_{ac}^2}\,\frac{\mathrm {d}m_{fc}^2}{m_{fc}^2}\times 2. \end{aligned}$$

Let us now compare with the expected behaviour of the probability in this region of phase-space i.e. $\mathrm {d}\mathscr {P}_{\mathrm {g}_\mathrm {f} \rightarrow \mathrm {g}\mathrm {g}}^{\mathrm {FI}}$, defined by

36 $$\begin{aligned} \mathrm {d}\mathscr {P}_{\mathrm {g}_\mathrm {f} \rightarrow \mathrm {g}\mathrm {g}}^{\mathrm {FI}}= \frac{C_A}{2}\,\frac{\alpha _{\mathrm {s}}}{2\pi } \, \frac{\mathrm {d}Q^2}{Q^2} \,\frac{1 + z^3}{1 - z} \, \mathrm {d}z. \end{aligned}$$

The FI kinematics (24) leads to

37 $$\begin{aligned} \mathrm {d}Q^2\,\mathrm {d}z = \frac{m_{\mathrm {dip}}^2-m_{bc}^2}{(m_{\mathrm {dip}}^2+m_{bc}^2)^2}\mathrm {d}m_{bc}^2\,\mathrm {d}m_{rc}^2, \end{aligned}$$

and

38 $$\begin{aligned} \mathrm {d}\mathscr {P}_{\mathrm {g}_\mathrm {f} \rightarrow \mathrm {g}\mathrm {g}}^{\mathrm {FI}}&=\frac{C_A}{2}\,\frac{\alpha _{\mathrm {s}}}{2\pi }\,\frac{\mathrm {d}m_{bc}^2\,\mathrm {d}m_{rc}^2}{m_{bc}^2} \nonumber \\&\quad \times \frac{(1 + z^3)(m_{\mathrm {dip}}^2-m_{bc}^2)}{m_{bc}^2(m_{\mathrm {dip}}^2+m_{bc}^2)+m_{rc}^2(m_{\mathrm {dip}}^2-m_{bc}^2)} \end{aligned}$$

The limit $m_{fc}^2 \rightarrow 0$ in the IF case corresponds to $m_{bc}^2 \rightarrow 0$ in the FI case. In this limit,

39 $$\begin{aligned} \mathrm {d}\mathscr {P}_{\mathrm {g}_\mathrm {f} \rightarrow \mathrm {g}\mathrm {g}}^{\mathrm {FI}}\sim \frac{C_A}{2}\,\frac{\alpha _{\mathrm {s}}}{2\pi }\,\frac{\mathrm {d}m_{bc}^2}{m_{bc}^2}\,\frac{\mathrm {d}m_{rc}^2}{m_{rc}^2}\,\left( 1+\left( 1-\frac{m_{rc}^2}{m_{\mathrm {dip}}^2}\right) ^3\right) . \end{aligned}$$

It clearly appears that the singularity structure of equation (39) is reproduced by the singularities present in equation (35), as desired. Moreover, the weight defined previously ensures that the probability defined in Eq. (35) comes with the right colour factor $C_A/2$. The extra non singular term $\left( 1+\left( 1-m_{rc}^2/m_{\mathrm {dip}}^2\right) ^3\right)$ in Eq. (39) actually approaches the value 2 when $m_{rc}^2 \rightarrow 0$, as in Eq. (35). This shows that gluon emissions off a $\mathrm {q}_{\mathrm {i}} - \mathrm {g}_{\mathrm {f}}$ dipole can be fully described by the probability $w_{\mathrm {q}_{\mathrm {i}} - \mathrm {g}_{\mathrm {f}}}\,\mathrm {d}\mathscr {P}_{\mathrm {q}\rightarrow \mathrm {q}_\mathrm {i} \mathrm {g}}^{\mathrm {IF}}$ only, without any double counting.

In terms of the usual variables, $p^2_{\perp \mathrm {evol}}= (1 - z)Q^2$ and *z*, one obtains

40 $$\begin{aligned} m_{fc}^2 = m_{\mathrm {dip}}^2\frac{1 - z}{z}, \quad m_{ac}^2 = Q^2 = \frac{p^2_{\perp \mathrm {evol}}}{1 - z}, \end{aligned}$$

which leads to the weight

41 $$\begin{aligned} w_{\mathrm {q}_{\mathrm {i}} - \mathrm {g}_{\mathrm {f}}} = \frac{m_{\mathrm {dip}}^2(1 - z)^2 + \frac{C_A}{2C_F}\,z\,p^2_{\perp \mathrm {evol}}}{m_{\mathrm {dip}}^2(1 - z)^2 + z\,p^2_{\perp \mathrm {evol}}}. \end{aligned}$$

The same procedure can be applied for the configuration $\mathrm {g}_{\mathrm {i}}$ - $\mathrm {q}_{\mathrm {f}}$ with the ISR $\mathrm {g}\rightarrow \mathrm {g}_{\mathrm {i}} \mathrm {g}$ and the FSR $\mathrm {q}_{\mathrm {f}}\rightarrow \mathrm {q}\mathrm {g}$. The weight here is

42 $$\begin{aligned} w_{\mathrm {g}_{\mathrm {i}} - \mathrm {q}_{\mathrm {f}}} = \frac{m_{\mathrm {dip}}^2(1 - z)^2 + \frac{2C_F}{C_A}\,z\,p^2_{\perp \mathrm {evol}}}{m_{\mathrm {dip}}^2(1 - z)^2 + z\,p^2_{\perp \mathrm {evol}}}. \end{aligned}$$

The two other dipole configurations ($\mathrm {q}_{\mathrm {i}} - \mathrm {q}_{\mathrm {f}}$ and $\mathrm {g}_{\mathrm {i}} - \mathrm {g}_{\mathrm {f}}$) do not need any correction since the two dipole ends there have the same flavour. Indeed, it has been shown that for a $\mathrm {q}_{\mathrm {i}} - \mathrm {q}_{\mathrm {f}}$ dipole, the first-order matrix element is explicitly reproduced by $\mathrm {d}\mathscr {P}_{\mathrm {q}\rightarrow \mathrm {q}\mathrm {g}}^{\mathrm {IF}}$ in the case of DIS. Therefore, the collinear limit and soft-gluon limit are reproduced without any double counting. Since this behaviour is universal, this feature can be easily generalized to other processes than DIS. The case of the $\mathrm {g}_{\mathrm {i}} - \mathrm {g}_{\mathrm {f}}$ dipole is completely similar to the $\mathrm {q}_{\mathrm {i}} - \mathrm {q}_{\mathrm {f}}$ one, only with a different colour factor.

#### Gluon polarization

The global-recoil shower implements two sources of azimuthal asymmetries: colour coherence and gluon plane polarization. The former is automatically included in the dipole formulation. That is, radiation off a $\{b + f\}$ dipole is assumed isotropic in azimuth, but after a boost to the event rest frame the radiation is biased in the azimuthal direction of *f*, even the one that would be thought of as ISR off the *b*.

The gluon polarization has to be considered separately, however. It has the effect of correlating the production and decay planes of a gluon. To be more specific, assume that parton *b* is a gluon, produced by $a \rightarrow b + c$, and branching by $b \rightarrow g + h$. In a frame where *b* is aligned along the *z* axis the angle $\varDelta \varphi = \varphi _c - \varphi _g$ should follow a distribution [39]

43 $$\begin{aligned} \frac{\mathrm {d}\mathscr {P}_{\varphi }}{\mathrm {d}\varphi } \propto 1 + c_{\mathrm {pol}}\cos (2\varDelta \varphi ), \end{aligned}$$

where $c_{\mathrm {pol}}$ depends on flavours and kinematics at the production and decay vertices of the gluon. (Note that $\varphi _a = \varphi _c$ and that $\varphi _h = \varphi _g + \pi$ gives the same $\cos (2\varDelta \varphi )$ as $\varphi _g$.)

There is some ambiguity which frame to use when *b* is set along the *z* axis. The natural choice, and the one we have used, is the $\{b + d\}$ rest frame, where *b* and the other-side incoming parton *d* are along the $\pm z$ axis. The disadvantage is that it may partly counteract the colour-coherence azimuthal asymmetry, induced by the boost from the $\{b + f\}$ rest frame. This problem would have been solved had the latter frame been used, where only gluon polarization gives azimuthal anisotropies. That frame does not have any obvious relation with the $b \rightarrow g + h$ decay, on the other hand, so would also be imperfect.

**Fig. 7:**
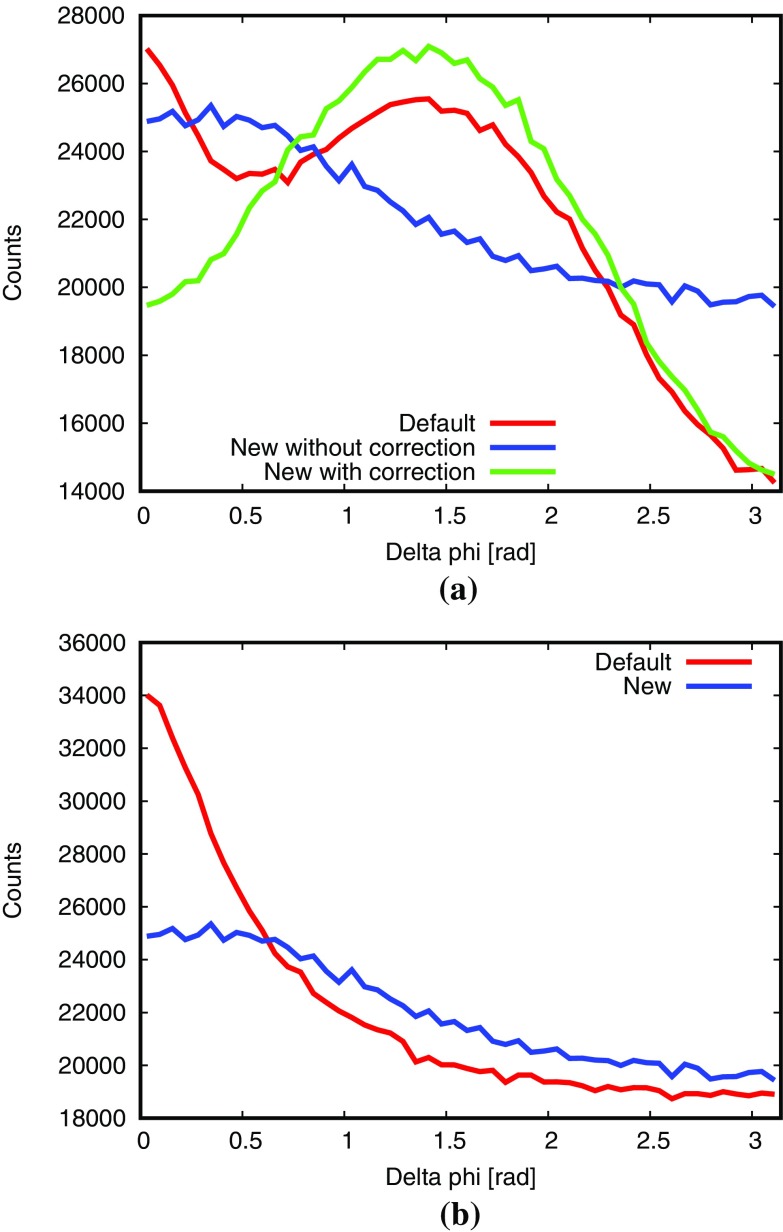
Histograms of the $\varDelta \varphi$ variable, as defined in the text, for $\gamma ^* / {\mathrm {Z}^0}$ production. In **a**, the red curve is for the old global-recoil scheme whereas blue and green are for the new dipole scheme with or without gluon polarization effects included. In **b**, the gluon polarization effects are removed so only the colour-coherence azimuthal asymmetries remain. Note the suppressed zero on the vertical axis

**Fig. 8:**
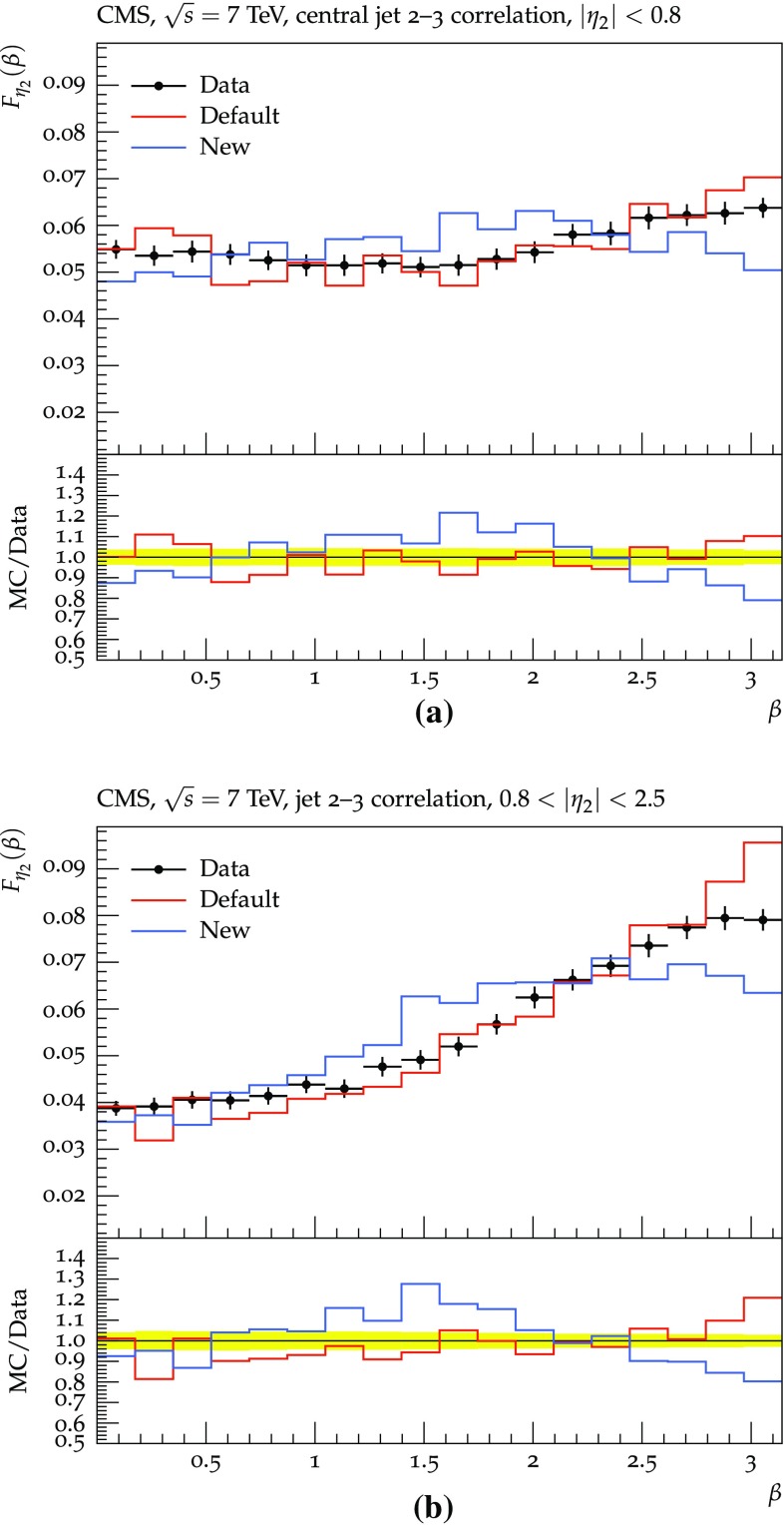
Angular correlation between the second and third jets measured by CMS for pp collisions at 7 TeV [44, 45]. The new and default procedures are compared, with all azimuthal asymmetries included. (Results are for QCD $2 \rightarrow 2$ events, generated with $\hat{p}_{\perp }> 60~\hbox {GeV}$, that survive the experimental selection.)

Results are given in Fig. 7a for the hard process $\mathrm {q}+ \overline{\mathrm {q}}\rightarrow \gamma ^* / {\mathrm {Z}^0}$ at 7 TeV. FSR, MPI and hadronization are turned off. The new procedure without polarization correction moderately favours small $\varDelta \varphi$, whereas the polarization effects favour $\varDelta \varphi \sim \pi /2$. Overall the latter curve is closer to the old default one, which is known to describe azimuthal asymmetries decently [1]. A significant difference arises for $\varDelta \varphi = 0$, however. This is due to the other source of azimuthal asymmetries, colour coherence, as shown in Fig. 7b, where gluon polarization effects have been switched off. The colour-coherence azimuthal distribution implemented in the default scheme clearly gives a stronger contribution for $\varDelta \varphi = 0$ than the ones automatically generated by the new scheme by the boost to the $\{b + d\}$ rest frame.

In Fig. 8, the difference between the two schemes is clearly visible in comparisons with data. The $\beta$ angle measures the distribution of the third jet around the second in three-jet QCD events, and is very sensitive to colour coherence effects [45]. The default procedure here gives a reasonably good description, while the new approach fares visibly worse. This is a surprising and unfortunate result, since the dipole approach ought to give the best description of colour-coherence azimuthal asymmetries. We have already seen that the gluon polarization effects can counteract the colour coherence ones, but for these distributions such effects appear to be small and do not offer an explanation. Therefore further studies will be necessary to understand this issue.

**Fig. 9:**
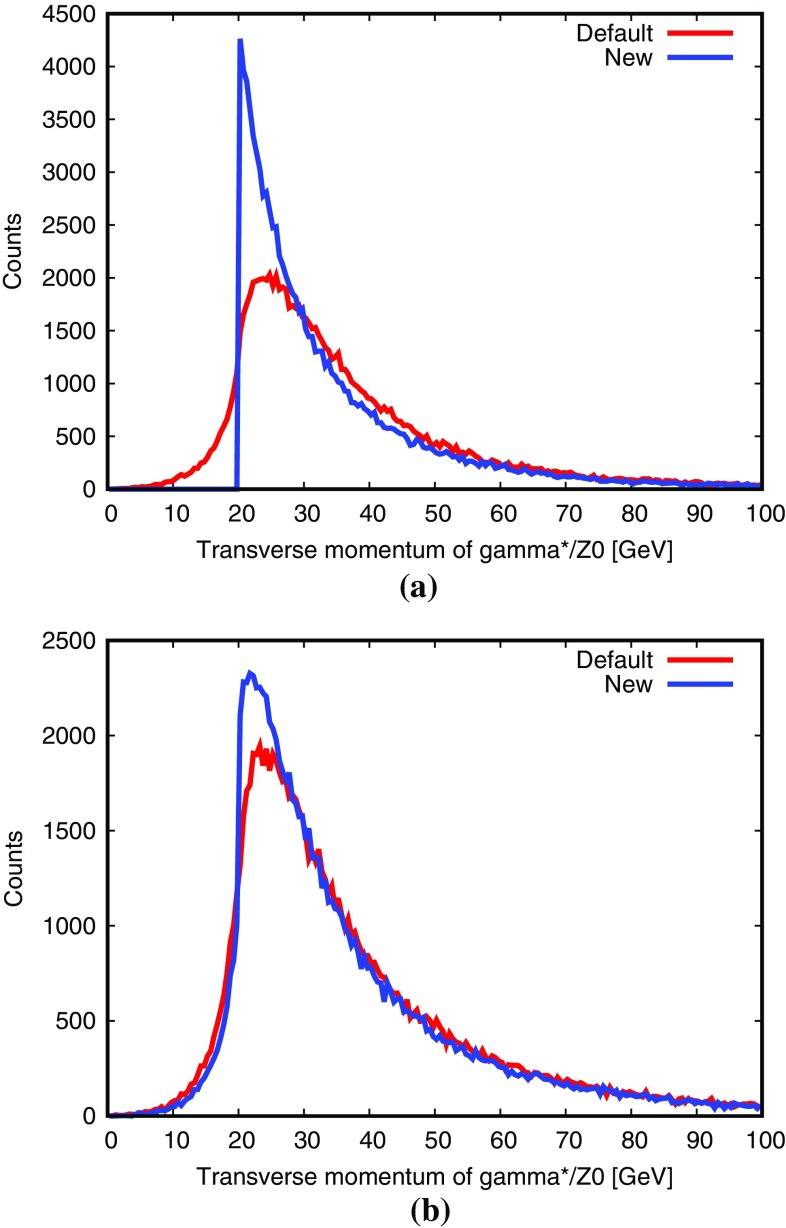
Transverse momentum of $\gamma ^* / {\mathrm {Z}^0}$ at 7 TeV LHC, with $\hat{p}_{\perp }> 20~\hbox {GeV}$ in the $2 \rightarrow 2$ process: **a** for $\mathrm {q}+ \overline{\mathrm {q}}\rightarrow \gamma ^* / {\mathrm {Z}^0}+ \mathrm {g}$, **b** for $\mathrm {q}+ \mathrm {g}\rightarrow \gamma ^* / {\mathrm {Z}^0}+ \mathrm {q}$. The new dipole approach is compared with the old default one. $p_{\perp }$ shifts due to primordial $k_{\perp }$ are not included here for simplicity

## Comparisons with data and with other approaches

### Gauge boson production

The process $\mathrm {q}+ \overline{\mathrm {q}}\rightarrow \gamma ^* / {\mathrm {Z}^0}+ \mathrm {g}$ allows a clean comparison between the dipole approach and the default global-recoil procedure. Indeed, the emission of a gluon off the $\mathrm {q}+ \overline{\mathrm {q}}$ dipole leads to the formation of two FI/IF dipoles, as shown in Fig. 4. Therefore, with the new scheme, the $p_{\perp }$ of a $\gamma ^* / {\mathrm {Z}^0}$ is fixed by the hard $2 \rightarrow 2$ process and is not altered by further emissions. The lower $p_{\perp }$ limit is then set by the choice of phase-space cuts. In contrast, with the global-recoil procedure, the $\gamma ^* / {\mathrm {Z}^0}$ $p_{\perp }$ can be increased, but also reduced, in consecutive branchings. Some typical results are given in Fig. 9a.

**Fig. 10:**
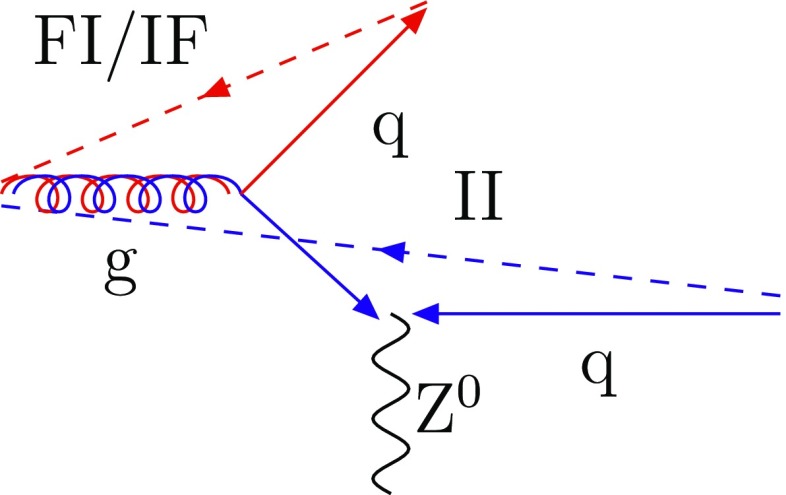
Colour flow for the process $\mathrm {q}+ \mathrm {g}\rightarrow \gamma ^* / {\mathrm {Z}^0}+ \mathrm {q}$. The dashed lines represent the colour lines stretched between the dipole ends

In Fig. 9b, the $\gamma ^* / {\mathrm {Z}^0}$ $p_{\perp }$ spectrum is also given for the process $\mathrm {q}+ \mathrm {g}\rightarrow \gamma ^* / {\mathrm {Z}^0}+ \mathrm {q}$. This process is interesting because it leads to the formation of one FI/IF dipole $\mathrm {g}_{\mathrm {i}}-\mathrm {q}_{\mathrm {f}}$ and one II dipole $\mathrm {g}_{\mathrm {i}}-\mathrm {q}_{\mathrm {i}}$, as illustrated in Fig. 10. Therefore, an emission off $\mathrm {g}_{\mathrm {i}}$ can be described either in the IF framework or in the II picture involving global recoils. In the first case, the $\gamma ^* / {\mathrm {Z}^0}$ will not get any recoil when the new scheme is used, but in the second it will. This is illustrated in Fig. 9b, where the new scheme now closer agrees with the older one.

**Fig. 11:**
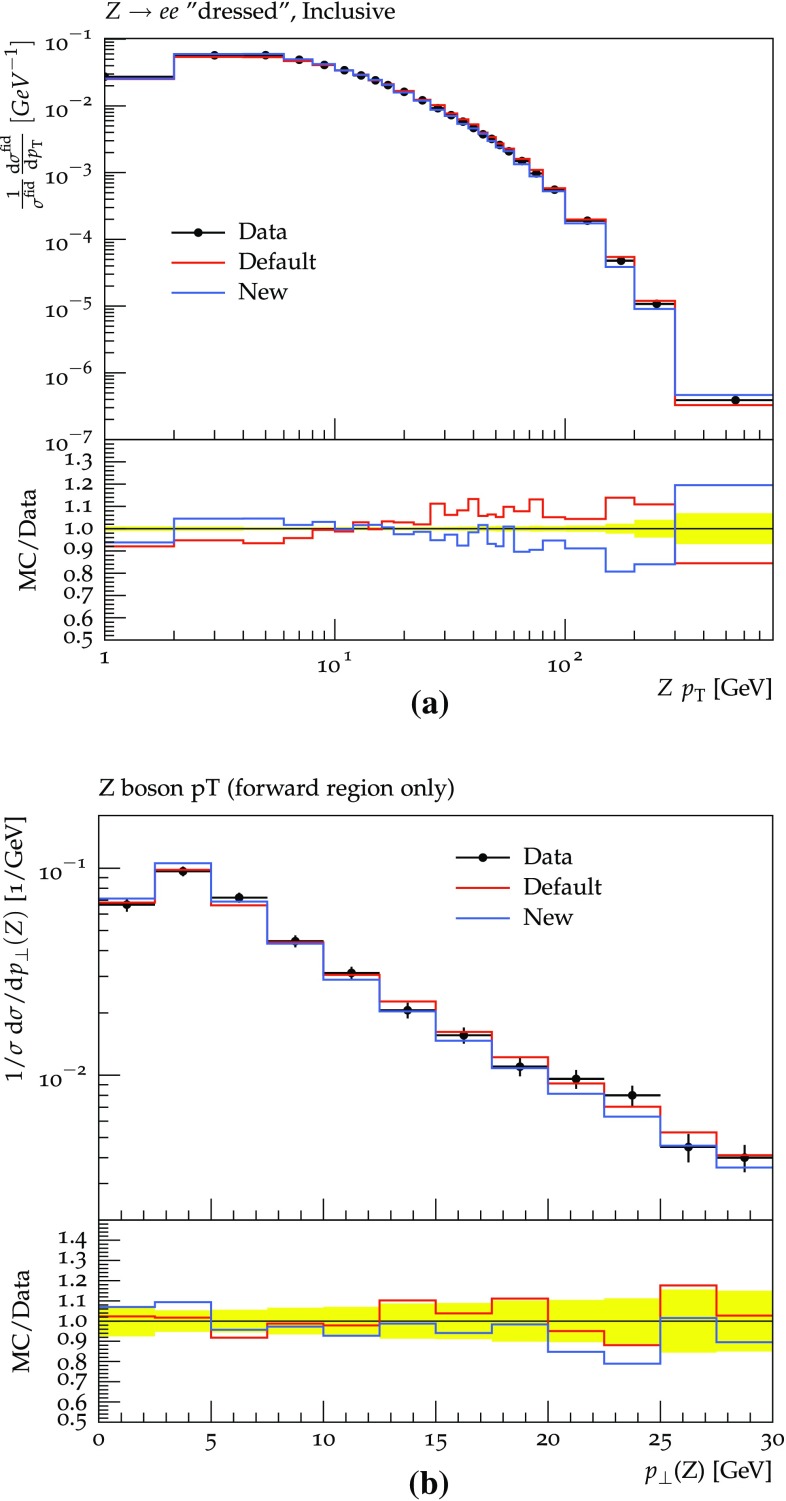
Comparison between the new and old schemes for the $\gamma ^* / {\mathrm {Z}^0}$ $p_{\perp }$ spectrum measured by **a** ATLAS for pp collisions at 7 TeV [44, 46], **b** D0 for p$\overline{\mathrm {p}}$ collisions at 1.96 TeV [44, 47]

The inclusive $2 \rightarrow 1$ $\gamma ^* / {\mathrm {Z}^0}$ production process, followed by showers, can be compared with experimental data. Results are shown in Fig. 11 for the $\gamma ^* / {\mathrm {Z}^0}$ $p_{\perp }$, compared with ATLAS [46] and D0 [47] data. The two shower procedures are here seen to lead to similar results, but a tendency can be noted that the new scheme gives a spectrum slightly shifted towards lower $p_{\perp }$ values, as could have been expected.

**Fig. 12:**
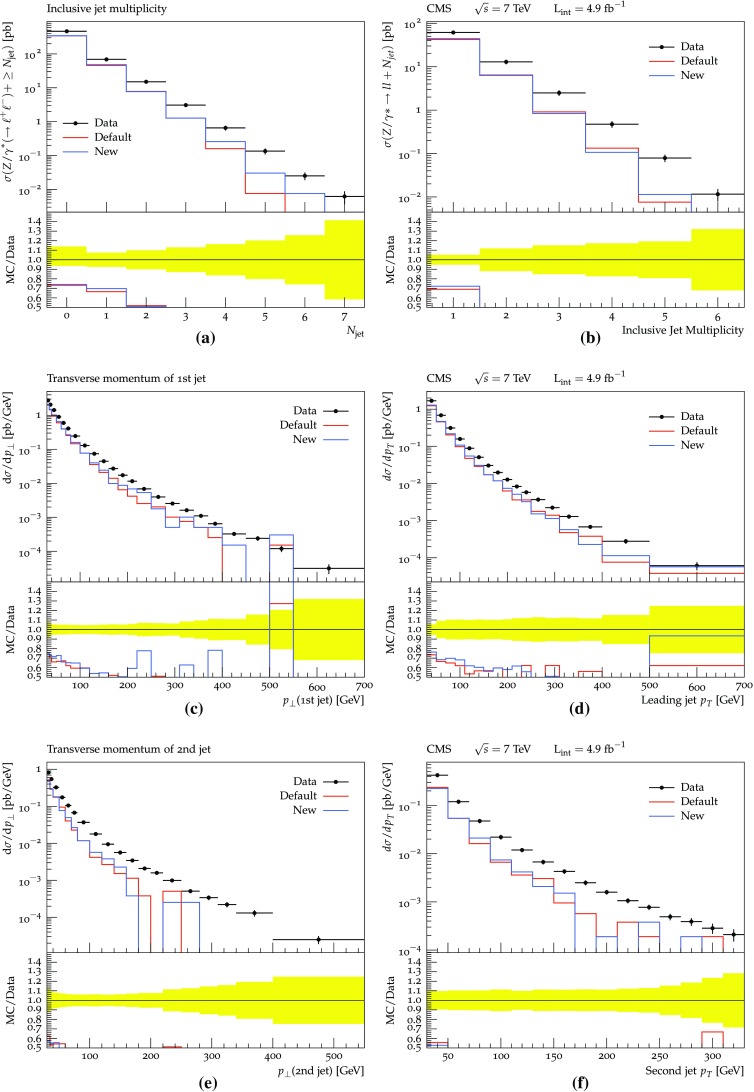
Jet production in inclusive $\gamma ^* / {\mathrm {Z}^0}$ events, as measured by ATLAS (**a**, **c** , **e**) and CMS (**b**, **d**, **f**), respectively, for pp collisions at 7 TeV [44, 48, 49]: **a**, **b** the number of jets, **c**, **d** $p_{\perp }$ of the first jet, **e**, **f** $p_{\perp }$ of the second jet

The production of $\gamma ^* / {\mathrm {Z}^0}$ is associated with jets. The multiplicity and $p_{\perp }$ spectra of such jets are given in Fig. 12, compared with ATLAS [48] and CMS [49] data. It is interesting to note that the new procedure seems to lead to a slightly higher jet activity, at least for the ATLAS jet definition, but overall differences are small. As before the high-multiplicity and high-$p_{\perp }$ tails are underestimated in the purely shower-based approach, so for a better description the need to inject information from higher-order matrix-elements remains unchanged.

### QCD jets

Another relevant area for comparisons is QCD jet production by $2 \rightarrow 2$ processes ($\mathrm {q}\mathrm {q}\rightarrow \mathrm {q}\mathrm {q}$, $\mathrm {g}\mathrm {g}\rightarrow \mathrm {g}\mathrm {g}$, $\mathrm {q}\mathrm {g}\rightarrow \mathrm {q}\mathrm {g}$, ...) followed by showers. These showers are evolved downwards from the $2 \rightarrow 2$ $\hat{p}_{\perp }$ scale, in order to avoid doublecounting.

**Fig. 13:**
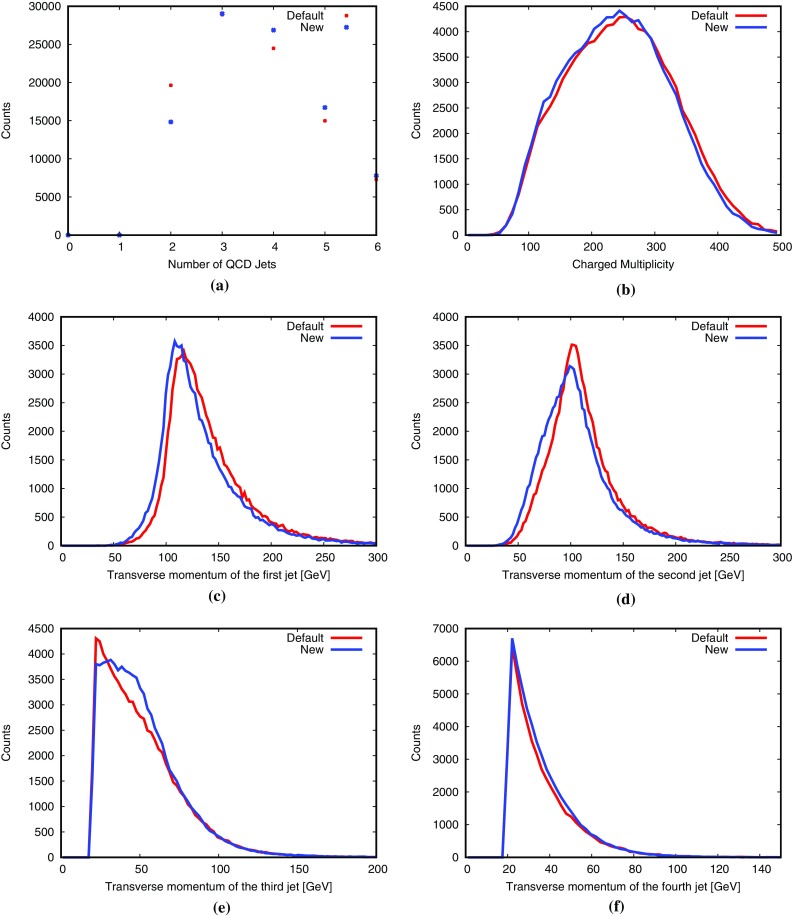
Comparison of shower algorithm results for the toy study described in the text: **a** number of QCD jets, **b** charged multiplicity, transverse momentum of the **c** first jet, **d** second jet, **e** third jet, **f** fourth jet

Again we begin by a toy study, for LHC with $\sqrt{s}=14$ TeV and $\hat{p}_{\perp }> 100~\hbox {GeV}$. Jets are defined by the anti-$k_{\bot }$ algorithm [50], with $R=0.7$ and $p_{\perp \mathrm {jet}} > 20~\hbox {GeV}$. Under these conditions the new procedure produces somewhat more jets than the default scheme, Fig. 13. Consistent with this the third and fourth jets (ordered by $p_{\perp }$) become harder, while the first two become softer. It is therefore slightly contradictory that the average charged multiplicity drops from 246 to 241 (with widths 85 and 82, respectively). Further studies will be needed to sort out why some distributions suggest more activity and others less.

**Fig. 14:**
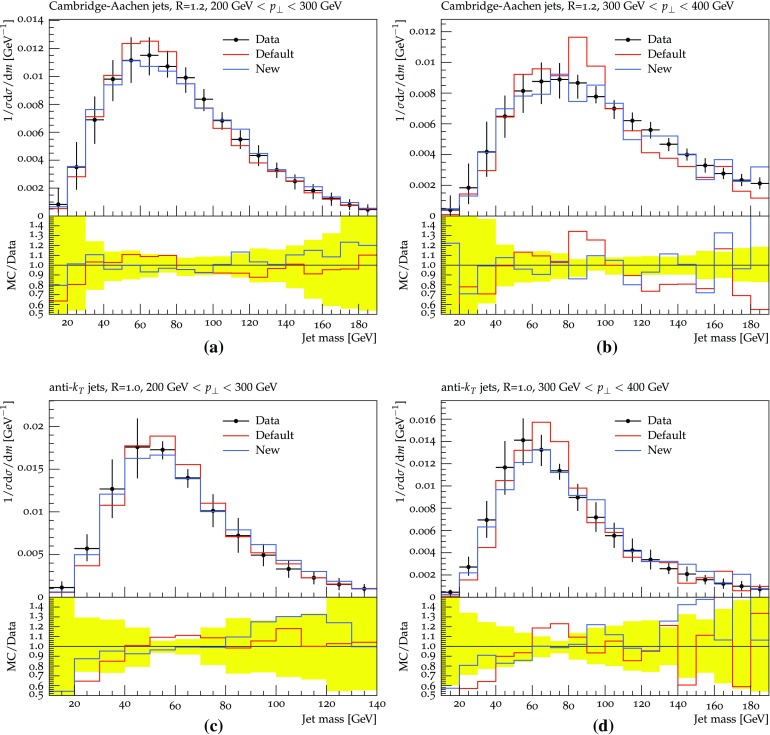
Jet mass spectra measured in QCD events by ATLAS for pp collisions at 7 TeV [44, 51]. The jets have been reconstructed with: **a**, **b** Cambridge-Aachen with $R=1.2$, **c**, **d** anti-$k_{\perp }$ with $R=1.0$

**Fig. 15:**
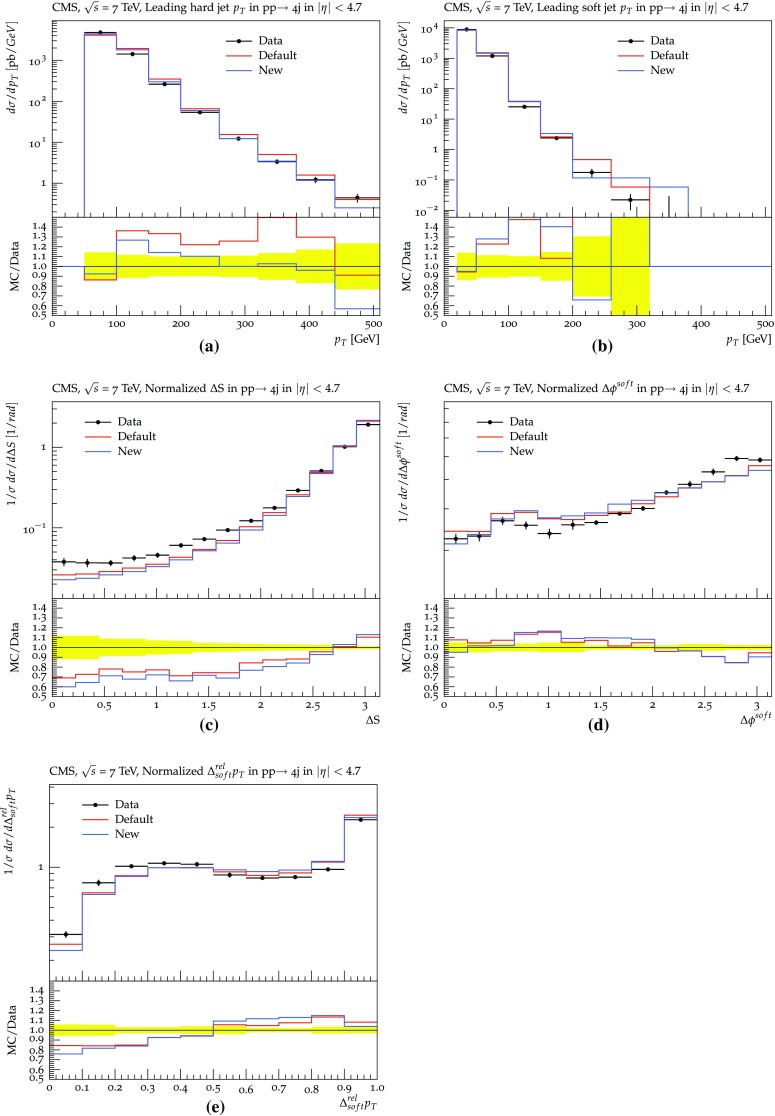
Four-jet cross-sections measured by CMS for pp collisions at 7 TeV [44, 52], as a function of several observables defined in [52]

Turning to real data, in Fig. 14 a few jet mass spectra measured by ATLAS [51] are presented. It can be seen that both shower procedures describe the data well, with some hints of improvements in the new scheme. In Fig. 15, the exclusive cross-section for the process pp $\rightarrow 4\, \mathrm {jets} + X$ is given as a function of several observables, as measured by CMS [52]. The $p_{\perp }$ spectra of the jets are well reproduced. For the plots involving angular variables, where the agreement is somewhat worse, the errors may be related to the same issues as already discussed for gluon polarization and colour coherence, but again ultimately point to the need for four-jet matrix-element input.

**Fig. 16:**
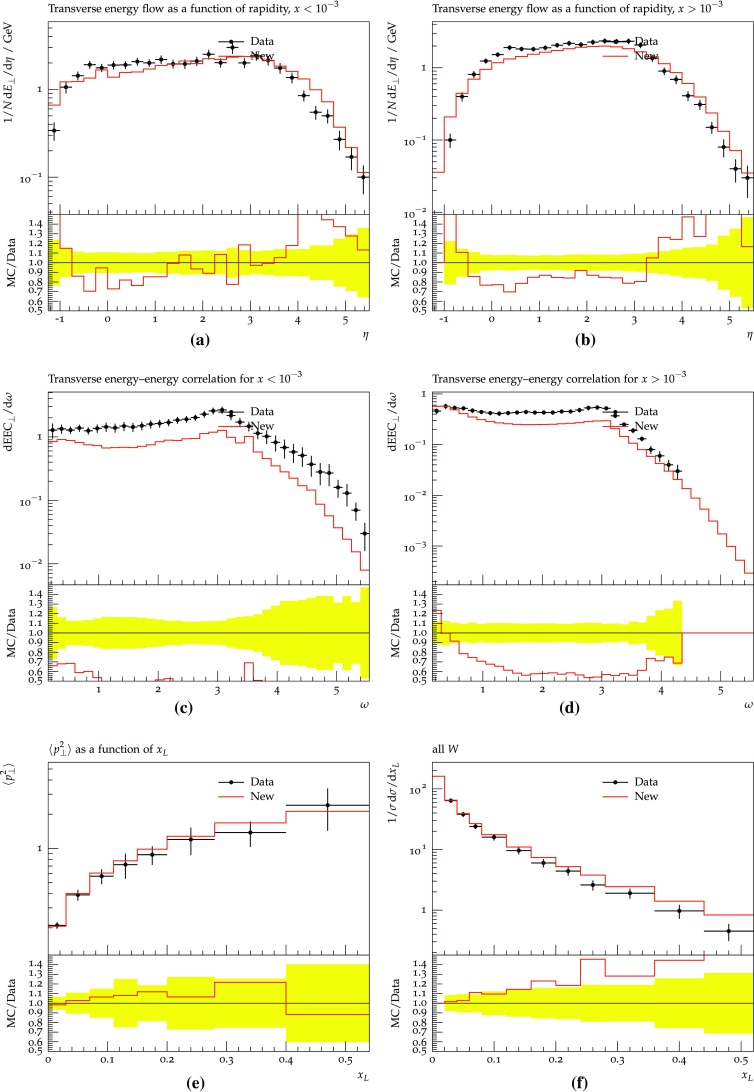
DIS events at HERA [44, 53]. The new scheme is compared with H1 data for $Q^2 > 40\,\mathrm {GeV}^2$. The definitions of the different observables can be found in [53]

### DIS

The new scheme gives the opportunity to study DIS. As shown before, the branching probability of an IF-type generates the full cross-section in the case of gluon emission. The dipole approach, applied to DIS, is then expected to reproduce the data decently. A first comparison has been done for HERA with a 820 GeV proton beam colliding a 26.7 GeV electron beam [53], Fig. 16. As can be seen, single-particle properties are reasonably well described, whereas the energy-energy correlation undershoots data. This could be studied further, e.g. by comparing with the Ariadne dipole model [13] which was known to give a very accurate description of these data. Unfortunately it cannot easily be combined with Pythia 8.

**Fig. 17:**
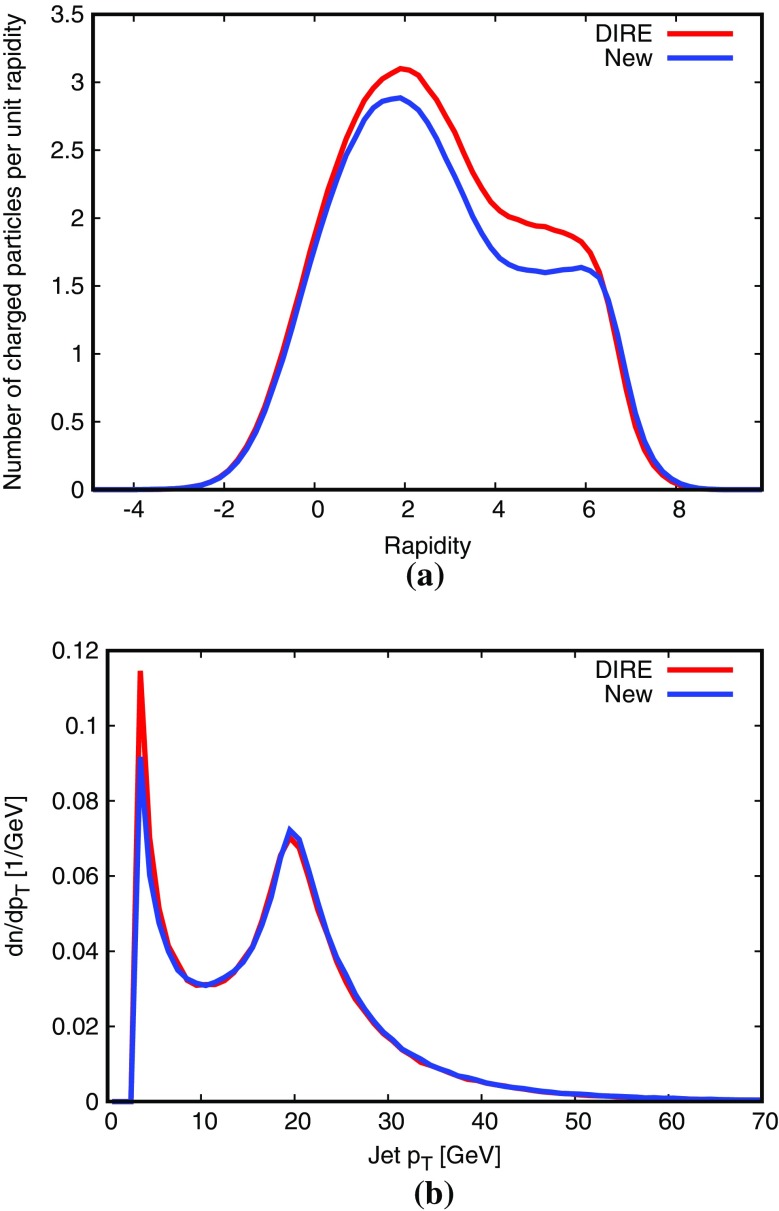
Comparison between the new approach and the Dire shower, HERA ep collisions with beam energies 27.5 and 920 GeV and $Q^2 > 400 \, \mathrm {GeV}^2$: **a** charged rapidity spectrum and **b** jet $p_{\perp }$ spectrum using the anti-$k_{\perp }$ algorithm with $R = 0.7$ and $p_{\perp \mathrm {min}}= 3~\hbox {GeV}$

An alternative for comparisons is instead offered by the Dire dipole shower program [20], which can be used as a plugin to Pythia 8, such that the shower algorithms is the only difference. Results turn out to be closely similar to each other in most variables, Fig. 17. It may be noted, however, that the charged multiplicity is somewhat higher in Dire, even though the jet rate is comparable. One reason is that Pythia by default uses a smooth dampening of ISR at small scales, similar to the one for MPIs [32, 54], while Dire has a lower sharp cutoff, giving it a larger partonic activity at small $p_{\perp }$ scales.

### Particle production rates

To finish, it is useful to reflect on one of the key features that distinguish the dipole from the global-recoil approach, that the amount of ISR depends on the invariant mass of the colour dipoles stretched out to the final state. To illustrate this, consider $\mathrm {q}+ \mathrm {q}' \rightarrow \mathrm {q}+ \mathrm {q}'$ with $\mathrm {q}\ne \mathrm {q}'$. Here only *t*-channel gluon exchange contributes, so colour flows from the incoming $\mathrm {q}$ to the outgoing $\mathrm {q}'$. A small quark scattering angle $\theta _{\mathrm {q}\mathrm {q}}$ (in the rest frame of the collision) thus corresponds to a large colour-flow scattering angle $\theta _{\mathrm {col}} = \theta _{\mathrm {q}\mathrm {q}'} = \pi - \theta _{\mathrm {q}\mathrm {q}}$, and vice versa. With cuts $\hat{m} = \sqrt{\hat{s}} > 500~\hbox {GeV}$ and $25< \hat{p}_{\perp }< 50~\hbox {GeV}$ for the hard $2 \rightarrow 2$ process, for LHC at 13 TeV, allowed scatterings split into one low-angle and one high-angle range. The total charged multiplicity for these cases is shown in Table 3. We see that, even without any showers or MPIs, the higher $\theta _{\mathrm {col}}$ range gives the larger multiplicity, because it implies higher-mass nonperturbative colour strings stretched between the scattered quarks and the beam remnants. The multiplicities come up when the old global showers are added, slightly more for higher $\theta _{\mathrm {col}}$: while the handling of the II dipole end is identical in the two cases, the FI one does contain a dependence on the colour dipole masses. In the new dipole shower the difference is much more pronounced, however. Even if the $p_{\perp \mathrm {evol}}$ scale of the shower evolution is constrained from above by the $p_{\perp }$ scale of the hard $2\rightarrow 2$ process in both cases, below that scale the phase space for emissions inside a dipole is (logarithmically) related to its mass, so a larger $\theta _{\mathrm {col}}$ opens up for more radiation.

In real life it is not feasible to tag whether a quark scattering occured at a small or a large angle, and for the dominant $\mathrm {g}+ \mathrm {g}\rightarrow \mathrm {g}+ \mathrm {g}$ processes it is not even a meaningful question to ask. There is only a small net remaining multiplicity difference between the old and new shower approaches if all QCD $2 \rightarrow 2$ processes at all angles are included, as we see in the third column of Table 3. A more differential picture can be obtained from the multiplicity dependence on the rapidity separation $|\varDelta y| \approx -2 \ln \tan (\theta /2)$ between the two hard jets, while still not distinguishing $\theta$ from $\pi - \theta$. And, unfortunately, both shower options show almost identically the same rise of the multiplicity with increasing $|\varDelta y|$, leaving no discriminating power.

**Table 3:** Average charged event multiplicity and the width of the multiplicity distribution without showers, or with the old global or new local showers. The first two columns are for $\mathrm {q}+ \mathrm {q}' \rightarrow \mathrm {q}+ \mathrm {q}'$ processes only, with cuts as described in the text, and the third for all $2 \rightarrow 2$ processes with $p_{\perp }> 25~\hbox {GeV}$. The last three columns are with MPIs also included, for events of increasing (average) hardness

Showering	No MPI			With MPI		
	low $\theta _{\mathrm {q}\mathrm {q}}$	High $\theta _{\mathrm {q}\mathrm {q}}$	All $\theta _{\mathrm {q}\mathrm {q}}$	minbias	$\hat{p}_{\perp }> 25~\hbox {GeV}$	$\hat{p}_{\perp }> 200~\hbox {GeV}$
	high $\theta _{\mathrm {col}}$	low $\theta _{\mathrm {col}}$	All $\theta _{\mathrm {col}}$			
No	$43 \pm 7$	$28 \pm 6$	$52 \pm 9$	$76 \pm 40$	$122 \pm 47$	$126 \pm 46$
Old global	$75 \pm 17$	$56 \pm 16$	$83 \pm 18$	$113 \pm 74$	$216 \pm 83$	$253 \pm 83$
New dipole	$82 \pm 17$	$40 \pm 10$	$82 \pm 18$	$110 \pm 72$	$209 \pm 79$	$248 \pm 80$

When MPIs are included the differences are slightly larger in absolute numbers, since each MPI gives its contribution to the net difference; see the last three columns of Table 3 for inclusive (nondiffractive) minimum-bias events, and jet events above two different $p_{\perp }$ thresholds for the hard process. Relative to the no-shower baseline it is still notable that the old and new showers add almost the same amount of extra activity. It may suggest that many semi-inclusive observables will also look rather similar, and that more specific observables will be needed to distinguish the two. Furthermore, the charged-multiplicity discrepancies presumably could be resolved by some modest retuning, e.g. a slightly larger $\alpha _{\mathrm {s}}$ for the new dipole showers. Such a retuning has not (yet) been done; at this stage of the studies it is useful to compare the two options under identical conditions.

## Summary and outlook

The dipole approach to showers is not new, and in that sense the study in this article does not provide anything fundamentally new. It does offer a few new insights, however, and access to a new useful tool.

One of the interesting aspects is the constraints imposed on the recoil kinematics. For a final–final dipole the emission recoil can be shared between the two dipole ends in many ways. But for an initial–final dipole a central constraint is that the initial incoming parton must be parallel with the beam axis. This enforces the same kinematics whether the process is viewed as that of final-state radiation with a recoil in the initial state or the other way around.

It could still be that the contributions from the initial- and final-state emissions would need to be added to obtain the complete initial–final dipole emission pattern. It would then be important to combine the two without gaps or doublecounting. The cleanest way is to compare with the radiation pattern in Deeply Inelastic Scattering, notably for the gluon-emission process, $\gamma ^* \mathrm {q}\rightarrow \mathrm {q}\mathrm {g}$. The pleasant surprise then is that initial-state emissions cover the full phase space on its own, with the correct denominator singularity structure and a finite numerator very close to the correct one. The final-state emissions do not give quite as simple an expression. A suitable reweighting could fix it, but the simple solution is to describe the full emission pattern by ISR and omit FSR altogether. Unfortunately the results are not as clean for gluon splittings, $\gamma ^* \mathrm {g}\rightarrow \mathrm {q}\overline{\mathrm {q}}$. This is no news; gluon splittings have never fitted well inside the dipole framework.

Some first comparisons with data have been presented in this article, and look promising, but not so very different from the old non-dipole approach. Partly this is because experimental procedures by necessity average over different topologies, thereby largely cancelling effects in the underlying dynamics, and partly because the old scheme approximated the boost effects by ISR azimuthal asymmetries. In fact, in some distributions the old approximate scheme gives larger effects than the new one does, and here data agrees better with the old one although the new one is theoretically better motivated.

It should be remembered, however, that the new dipole framework has not yet been tuned, but is based on the existing default tune for the old scheme, so disagreements were to be expected. Some difference thus may be tuned away, but others may remain. Furthermore, no attempt has been made to include matching and merging with higher-order matrix elements [1]. In such a more complete framework the difference between alternative showers are partly masked, since the showers then are not providing the hard topologies. The ordering of emissions and the Sudakov factors that go with that do depend on the shower algorithm, however, so the possibility to compare different algorithms is useful to assess uncertainties. One may also want to combine global and local recoils by what technically is most convenient for the matching and merging schemes, similarly to what is already available for FSR.

The new algorithm has been implemented in Pythia, and will soon be publicly available. This will allow more detailed comparisons to be made than the ones presented in this article. Comparisons with LHC data will here be the main application, needless to say. But it will also open up for DIS studies, which could not be done with Pythia 8 previously, except by linking to the Dire shower [20]. Do note, however, that currently QED emission is not included. The $\mathrm {e} + \mathrm {q}\rightarrow \mathrm {e} + \mathrm {q}$ process implies quadrupole radiation, that could be approximated by a sum of dipoles. This is another example where further studies and extensions should follow.

In summary, our new dipole-based algorithm for ISR offers an interesting alternative to the existing one. The new code can stand on its own right away for a number of interesting studies, but to realize the full potential it may require some further extensions.

## References

[1] Buckley A (2011). General-purpose event generators for LHC physics. Phys. Rept..

[2] Particle Data Group Collaboration, C. Patrignani et al., Review of Particle Physics, Chin. Phys. C **40**(10), 100001 (2016)

[3] Gribov VN, Lipatov LN (1972). Deep inelastic e p scattering in perturbation theory. Sov. J. Nucl. Phys..

[4] Altarelli G, Parisi G (1977). Asymptotic freedom in parton language. Nucl. Phys. B.

[5] Dokshitzer YL (1977). Calculation of the Structure Functions for Deep Inelastic Scattering and e+ e- Annihilation by Perturbation Theory in Quantum Chromodynamics. Sov. Phys. JETP.

[6] Marchesini G, Webber BR (1984). Simulation of QCD jets including soft gluon interference. Nucl. Phys. B.

[7] Marchesini G, Webber BR (1988). Monte Carlo simulation of general hard processes with coherent QCD radiation. Nucl. Phys. B.

[8] Gieseke S, Stephens P, Webber B (2003). New formalism for QCD parton showers. JHEP.

[9] Bähr M (2008). Herwig++ physics and manual. Eur. Phys. J. C.

[10] Bellm J (2016). Herwig 7.0/Herwig++ 3.0 release note. Eur. Phys. J..

[11] Gustafson G (1986). Dual description of a confined color field. Phys. Lett. B.

[12] Gustafson G, Pettersson U (1988). Dipole formulation of QCD cascades. Nucl. Phys. B.

[13] Lönnblad L (1992). ARIADNE version 4: a program for simulation of QCD cascades implementing the color dipole model. Comput. Phys. Commun..

[14] ’t Hooft G (1974). A planar diagram theory for strong interactions. Nucl. Phys.

[15] Schumann S, Krauss F (2008). A parton shower algorithm based on Catani-Seymour dipole factorisation. JHEP.

[16] Gleisberg T, Hoeche S, Krauss F, Schonherr M, Schumann S, Siegert F, Winter J (2009). Event generation with SHERPA 1.1. JHEP.

[17] Hoeche S, Schumann S, Siegert F (2010). Hard photon production and matrix-element parton-shower merging. Phys. Rev. D.

[18] Giele WT, Kosower DA, Skands PZ (2008). A simple shower and matching algorithm. Phys. Rev. D.

[19] Fischer N, Prestel S, Ritzmann M, Skands P (2016). Vincia for hadron colliders. Eur. Phys. J. C.

[20] Höche S, Prestel S (2015). The midpoint between dipole and parton showers. Eur. Phys. J. C.

[21] Plätzer S, Gieseke S (2011). Coherent parton showers with local recoils. JHEP.

[22] Catani S, Seymour MH (1997). A general algorithm for calculating jet cross sections in NLO QCD. Nucl. Phys. B.

[23] Sjöstrand T, Skands PZ (2005). Transverse-momentum-ordered showers and interleaved multiple interactions. Eur. Phys. J. C.

[24] Sjöstrand T, Mrenna S, Skands PZ (2006). PYTHIA 6.4 physics and manual. JHEP.

[25] Sjöstrand T, Ask S, Christiansen JR, Corke R, Desai N, Ilten P, Mrenna S, Prestel S, Rasmussen CO, Skands PZ (2015). An introduction to PYTHIA 8.2. Comput. Phys. Commun..

[26] Carli T, Gehrmann T, Hoeche S (2010). Hadronic final states in deep-inelastic scattering with Sherpa. Eur. Phys. J. C.

[27] Kato K, Munehisa T (1991). NLLjet : a Monte Carlo code for e+ e- QCD jets including next-to-leading order terms. Comput. Phys. Commun..

[28] Li HT, Skands P (2017). A framework for second-order parton showers. Phys. Lett. B.

[29] S. Höche, S. Prestel, Triple collinear emissions in parton showers. arXiv:1705.00742 [hep-ph]

[30] S. Höche, F. Krauss, S. Prestel, Implementing NLO DGLAP evolution in Parton Showers. arXiv:1705.00982 [hep-ph]

[31] Plätzer S, Sjödahl M (2012). Subleading $$N_c$$ improved parton showers. JHEP.

[32] Corke R, Sjöstrand T (2011). Interleaved parton showers and tuning prospects. JHEP.

[33] Sudakov VV (1956). Vertex parts at very high-energies in quantum electrodynamics. Sov. Phys. JETP.

[34] Sjöstrand T (1985). A model for initial state parton showers. Phys. Lett. B.

[35] Bengtsson M, Sjöostrand T (1987). Coherent parton showers versus matrix elements: implications of PETRA—PEP data. Phys. Lett. B.

[36] Norrbin E, Sjöstrand T (2001). QCD radiation off heavy particles. Nucl. Phys.

[37] B. Cabouat, Parton Shower Algorithms—Possible Improvements, Master’s thesis, Lund U. (2017)

[38] Miu G, Sjöstrand T (1999). $$W$$ production in an improved parton shower approach. Phys. Lett.

[39] Webber BR (1986). Monte Carlo simulation of hard hadronic processes. Ann. Rev. Nucl. Part. Sci..

[40] Dokshitzer YL, Diakonov D, Troian SI (1980). Hard processes in quantum chromodynamics. Phys. Rept..

[41] ATLAS Collaboration, G. Aad et al., Measurement of the electroweak production of dijets in association with a Z-boson and distributions sensitive to vector boson fusion in proton-proton collisions at $$\sqrt{s} = 8\ \text{TeV}$$ using the ATLAS detector, JHEP**04** 031, (2014). arXiv:1401.7610 [hep-ex]

[42] Alwall J, Frederix R, Frixione S, Hirschi V, Maltoni F, Mattelaer O, Shao HS, Stelzer T, Torrielli P, Zaro M (2014). The automated computation of tree-level and next-to-leading order differential cross sections, and their matching to parton shower simulations. JHEP.

[43] Andersson B, Gustafson G, Lönnblad L (1990). Gluon splitting in the color dipole cascades. Nucl. Phys. B.

[44] Buckley A, Butterworth J, Lonnblad L, Grellscheid D, Hoeth H, Monk J, Schulz H, Siegert F (2013). Rivet user manual. Comput. Phys. Commun..

[45] Collaboration CMS, Chatrchyan S (2014). Probing color coherence effects in pp collisions at $$\sqrt{s}=7$$ TeV. Eur. Phys. J. C.

[46] **ATLAS** Collaboration, G. Aad et al., Measurement of the $$Z/\gamma ^{*}$$ boson transverse momentum distribution in $$pp$$ collisions at $$\sqrt{s} = 7\ \text{ TeV }$$ with the ATLAS detector. JHEP **09**, 145 (2014). arXiv:1406.3660 [hep-ex]

[47] D0 Collaboration, V. M. Abazov et al., Measurement of the shape of the boson transverse momentum distribution in $$p \bar{p} \rightarrow Z / \gamma ^{*} \rightarrow e^+ e^- + X$$ events produced at $$\sqrt{s}=1.96-\text{ TeV }$$, Phys. Rev. Lett. **100**, 102002 (2008). arXiv:0712.0803 [hep-ex] 10.1103/PhysRevLett.100.102002 18352175

[48] ATLAS Collaboration Collaboration, G. Aad et al., Measurement of the production cross section of jets in association with a Z boson in pp collisions at $$\sqrt{s} = 7\ \text{ TeV }$$ with the ATLAS detector. JHEP **1307** 032, (2013). arXiv:1304.7098 [hep-ex]

[49] Collaboration CMS, Khachatryan V (2015). Measurements of jet multiplicity and differential production cross sections of $$Z +$$ jets events in proton-proton collisions at $$\sqrt{s}=7$$ TeV. Phys. Rev. D.

[50] Cacciari M, Salam GP, Soyez G (2008). The anti-$$k_{\perp }$$ jet clustering algorithm. JHEP.

[51] ATLAS Collaboration Collaboration, G. Aad et al., Jet mass and substructure of inclusive jets in $$\sqrt{s}=7$$ TeV $$pp$$ collisions with the ATLAS experiment, JHEP **1205** 128, (2012). arXiv:1203.4606 [hep-ex]

[52] CMS Collaboration Collaboration, S. Chatrchyan et al., Measurement of four-jet production in proton-proton collisions at $$\sqrt{s}=7$$ TeV. arXiv:1312.6440 [hep-ex]

[53] H1 Collaboration, I. Abt et al., Energy flow and charged particle spectrum in deep inelastic scattering at HERA, Z. Phys. C **63**, 377–390 (1994)

[54] Sjöstrand T, van Zijl M (1987). A Multiple Interaction Model for the Event Structure in Hadron Collisions. Phys. Rev. D.

